# miRNA‐29 regulates epidermal and mesenchymal functions in skin repair

**DOI:** 10.1002/1873-3468.70051

**Published:** 2025-04-25

**Authors:** Lalitha Thiagarajan, Rosa Sanchez‐Alvarez, Chiho Kambara, Poojitha Rajasekar, Yuluang Wang, François Halloy, Jonathan Hall, Hans‐Jürgen Stark, Iris Martin, Petra Boukamp, Svitlana Kurinna

**Affiliations:** ^1^ Division of Cell Matrix Biology and Regenerative Medicine, School of Biological Sciences University of Manchester UK; ^2^ Division of Respiratory Medicine University of Nottingham UK; ^3^ Department of Chemistry and Applied Biosciences, Institute of Pharmaceutical Sciences ETH Zurich Switzerland; ^4^ German Cancer Research Center Heidelberg Germany

**Keywords:** adhesion, fibroblasts, keratinocytes, miRNA, miRNA‐CLIP, mRNA

## Abstract

MicroRNAs (miRNAs) control organogenesis in mammals by inhibiting translation of mRNA. Skin is an excellent model to study the role of miRNAs in epidermis and the mesenchyme. Previous research demonstrated miRNA‐29 family functions in skin; however, the mRNA targets and the downstream mechanisms of miRNA‐29‐mediated regulation are missing. We used the miRNA crosslinking and immunoprecipitation method to find direct targets of miRNA‐29 in keratinocytes and fibroblasts from human skin. miRNA‐29 inhibition using modified antisense oligonucleotides in 2D and 3D cultures of keratinocytes and fibroblasts enhanced cell‐to‐matrix adhesion through autocrine and paracrine mechanisms of miRNA‐29‐dependent tissue growth. We reveal a full transcriptome of human keratinocytes with enhanced adhesion to the matrix, which supports regeneration of the epidermis and is regulated by miRNA‐29.

**Impact statement**

The functions of small, therapeutically targetable microRNA molecules identified in our study can provide a new approach to improve wound healing by restoring and enhancing the inner molecular mechanisms of a cell and its surrounding matrix. We also provide a plethora of new mRNA targets for follow‐up studies of cell adhesion and extracellular matrix formation in humans.

## Graphical abstract

miRNA‐29 inhibits cell‐to‐cell and cell‐to‐matrix adhesion by silencing mRNA targets. Adhesion is controlled by complex interactions between many types of molecules coded by mRNAs. This is crucial for keeping together the layers of the skin and for regenerating the skin after wounding. Binding of miRNA‐29 with antisense nucleotides (ASO) rescues silencing of the mRNA, activating mechanisms to form new adhesions.

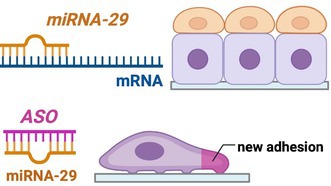

## Abbreviations

**3′UTR**, 3′ untranslated region

**abc**, ASO against miRNA‐29a/b/c

**abm**, miRNA‐29a/b1 mimic

**AGO2**, Argonaute protein 2

**aSMA**, alpha smooth muscle actin

**ASO**, antisense oligo

**BM**, basal lamina

**BP**, biological pathways

**BrdU**, bromodeoxyuridine

**CC**, cellular compartments

**CEACAM**, carcinoembryonic antigen cell adhesion molecule

**CLIP**, crosslinked immunoprecipitation

**COL4**, collagen 4

**DAVID**, the Database for annotation, visualization, and integrated discovery

**DCR8**, DiGeorge syndrome critical region gene 8

**DF**, dermal fibroblasts

**ECM**, extracellular matrix

**EGF**, epithelial growth factor

**EMT**, epithelial to mesenchymal transition

**FERMT2**, FERM domain containing kindlin 2

**FISH**, fluorescent *in situ* hybridization

**FN**, fibronectin

**GO**, gene onthology

**HFK**, hair follicular keratinocytes

**ICAM**, intercellular adhesion molecule

**IFK**, interfollicular keratinocytes

**IP**, immunoprecipitation

**IPA**, Ingenuity Pathway Analysis

**ITGA6**, integrin alpha 6

**ITGB1**, integrin beta 1

**L1CAM**, L1 cell adhesion molecule

**MF**, molecular functions

**miRNA**, microRNA

**NGS**, next‐generation sequencing

**nsa**, non‐specific ASO

**nsm**, non‐specific mimic

**PCA**, principal component analysis

**pre‐miRNAs**, precursor miRNAs

**qRT‐PCR**, quantitative real‐time polymerase chain reaction

**RIP**, RNA immunoprecipitation

**RISC**, RNA‐induced silencing complex

**SE**, skin equivalent

**SPARC**, secreted protein acidic and rich in cysteine

**TGF‐β**, transforming growth factor beta

MicroRNAs (miRNAs) are short non‐coding RNAs that regulate gene silencing by guiding Argonaute (AGO) proteins to miRNA target sites in the 3' untranslated region (UTR) of mRNAs, thus suppressing protein translation [1]. AGO2 is the most highly expressed AGO protein that cleaves an mRNA target fully complementary to the miRNA [2]. miRNA biogenesis starts with the RNA polymerase II – mediated transcription of primary miRNAs (pri‐miRNAs), which are processed into precursor miRNAs (pre‐miRNAs) by the DCR8/Drosha complex and into mature miRNA duplexes by the RNase III enzyme Dicer. Finally, one strand of the mature miRNA is loaded into AGO, whereas the other strand is discarded [2].

miRNAs regulate cell plasticity and are especially important in defining cell fate during differentiation and regeneration of tissues and organs [2]. miRNA crosslinking and immunoprecipitation (miRNA‐CLIP) can identify miRNA targets in a cell‐type‐specific manner by directly crosslinking and precipitating functional AGO2 complexes, the RNA‐induced silencing complex (RISC) of miRNA‐mRNA bound by the “seed”‐sequence match [3, 4, 5]. If coupled with the gain‐ and loss‐of‐function experiments, this method has the potential to elicit the function of a miRNA in the most direct, functional way, identifying both new miRNA‐mRNA pairs and placing the known mRNA targets into biological pathways regulated by the miRNA.

Skin regeneration is an excellent model of adult tissue‐ and organogenesis, which is tightly controlled at both the transcriptional and translational levels by epigenetic factors, including miRNAs [6]. The formation of the epidermis during the development and regeneration of the skin implicates strong and rapid adhesion of the epidermal cells (keratinocytes) to the basal lamina, continuous proliferation and differentiation of keratinocytes into the suprabasal layers [7]. Small excisional wounds created on the mouse dorsum close within a week, regenerating epidermis very rapidly through the proliferation and migration of the surrounding intact cells [8]. Inside the wound, the newly formed epidermis has more layers compared to unwounded skin and in this, resembles human epidermis. Recent advances in treating severe skin conditions with autologous transgenic therapies emphasize the importance of modulating the molecular mechanisms involved in repair [9]. However, the mechanisms of miRNA activity toward cell‐specific transcriptomes in the skin epidermis and in the underlying dermis are largely missing. These co‐cultures of keratinocytes and fibroblasts in 3D, also known as organotypic skin cultures [10], resemble the growth and stratification of human epidermis when exposed to the air‐liquid interphase and can be grafted onto open wounds in rodents [11] and in human patients [9].

*In silico* and *in vitro* analyses can only partially identify the *in vivo* functions of miRNAs, because these functions depend on the expression of miRNA‐RNA pairs in the epidermal and dermal cells during the specific stage of their cell cycle or differentiation. We demonstrated that miRNA‐CLIP allows unbiased identification of mRNA targets in epidermal cells (keratinocytes) and in dermal fibroblasts using RNA sequencing, which we then combined with the functional assays, specific for keratinocytes and fibroblasts. We successfully implemented this approach to unveil the complex targetome of miRNA‐29 family of miRNA‐29a/b/c, which are known to function in developing normal cells [12, 13, 14] and in cancer [15]. In mammals, the miRNA‐29 family is encoded by two genes carrying four precursor sequences, pre‐miRNA‐29a, pre‐miRNA‐29b1, pre‐miRNA‐29b2, and pre‐miRNA‐29c, processed into three functional miRNAs, miRNA‐29a‐3p, miRNA‐29b‐3p, and miRNA‐29c‐3p with a common “seed” sequence. We and others previously detected very little endogenous activity for miRNA‐29b [12, 16, 17], suggesting that most of miRNA‐29 functions are exerted by miRNA‐29a and miRNA‐29c, which differ by only one nucleotide outside of the “seed” region. The targetome of the miRNA‐29 family includes mRNAs that regulate cell cycle [18], fibrosis [19], apoptosis [20], and cell differentiation [21]. Our results are supported by the reports on miRNA‐29 functions in different organs and tissues and suggest a role for miRNA‐29 in regenerating keratinocytes [12, 14, 16] and in dermal fibroblasts [19, 22]. We used human skin as a model to unveil the cell‐specific miRNA‐29 targetome of keratinocytes and fibroblasts during their growth and differentiation in 2D and 3D models of human skin *ex vivo*. We tested the psoralen‐biotin‐modified precursor of miRNA‐29, which was processed by the Dicer into an active miRNA‐29 and incorporated into to a functional RISC. We then proceeded with the miRNA‐29‐CLIP experiments in primary human keratinocytes and fibroblasts, which revealed a function of miRNA‐29 in cell adhesion and extracellular matrix (ECM). We confirmed this by identifying specific regulators of rapid keratinocyte adhesion, a function essential for clonal expansion in regenerative medicine of the skin [9]. Altogether, we reported the transcriptome directly regulated by miRNA‐29 in human hair follicle, interfollicular keratinocytes, and in dermal fibroblasts. Using antisense miRNA‐29 oligonucleotides as inhibitors of miRNA‐29 in the cells, we discovered previously unreported endogenous functions of miRNA‐29 in basal keratinocyte adhesion, fibroblast proliferation, and the formation of the ECM, which in turn, enhanced the rapid adhesion of keratinocytes through paracrine mechanisms. We identified FERMT2, SPARC, and COL4 as master regulators of keratinocyte adhesion and ECM deposition downstream of miRNA‐29. Our results demonstrated that inhibition of miRNA‐29 in keratinocytes and fibroblasts can be used to improve skin regeneration.

## Materials and methods

### Mouse maintenance and experimentation

C57Bl6 mice were bred and maintained under specific pathogen‐free conditions at the University of Manchester animal facility, where mice obtained food and water *ad libitum*. Mouse maintenance and experiments were approved by the UK Home Office, project license PDBD459C2. Male and female mice at the age of 8–10 months were used for all experiments. Analgesia (buprenorphine, 0.05–0.1 mg·kg^−1^) was administered subcutaneously prior to anesthesia, as well as following the initial wound biopsy, as directed by the veterinary surgeon. Two 6 mm wounds were introduced into the dorsum of each mouse using a 6 mm sterile biopsy hole punch (Stiefel, Brentford, UK). Wounds were collected using an 8 mm biopsy punch post‐mortem. Only mice with hair follicles (HFs) in the telogen phase were wounded and analyzed.

### Human skin and wound samples

The use of human chronic wound samples was approved by the NHS Health Research Authority from the Complexwounds@manchester biobank with patient consent under protocol REC#18/NW/0847. The study methodologies conformed to the standards set by the Declaration of Helsinki. The experiments were undertaken with the understanding and written consent of each subject. The samples used for this study were freshly collected from surgically debrided wounds. Laminin‐5 staining was performed following the protocol for fluorescent *in situ* hybridization for miRNA‐29 and scrambled controls described in detail below and in [12].

### Cell culture and transfection of oligonuclotides

Primary human cells, which were isolated from neonatal foreskin representing interfollicular keratinocytes (IFK) and dermal fibroblasts (DF), were obtained from PeloBiotech and maintained in serum‐free keratinocyte growth media (PeloBiotech, Martinsried, Germany) at 37 °C, 5% CO_2_ on Coating Matrix Kit Protein (Gibco, Altrincham, UK) coated flasks. Human follicular keratinocytes (HFK) were isolated from the hair of healthy donors and maintained under the same conditions. The cells were used at passage 3–4 in all experiments. DF were maintained in fibroblast growth medium kit (PeloBiotech) at 37 °C, 5% CO_2_. The miRNA‐29‐CLIP probe 409 was diluted in Optimem (Life Technologies, Paisley, UK) to a final concentration of 7.5 nm (keratinocytes) or 5 nm (fibroblasts) and transfected using RNAiMax (Life Technologies) to four 15‐cm dishes of cells at 90% confluency, which were then incubated for 24 h. A mock transfection was set up with the same conditions as above without the probe. In our initial experiments, higher miRNA‐29a/b1 expression was detected in keratinocytes than in fibroblasts. Due to this difference in endogenous levels of miRNA‐29 in HFK, IFK, and DF, we used low concentrations of the probe of 7.5 nm for keratinocytes (IFK and HFK) and 5 nm for fibroblasts (DF), which would not elicit a strong gain‐of‐function response.

For stem‐loop miRNA mimics and single‐stranded antisense oligonucleotide (inhibitors) transfections, the cells were seeded at 70–80% and 50–60% confluence, respectively (day 0) and incubated overnight at 37 °C, 5% CO_2_. On day 1, the cells were transfected with miRNA mimics or miRNA inhibitors at a final concentrations of 50 and 200 nm, respectively, using Lipofectamine RNAiMAX (Thermo Fisher Scientific, Altrincham, UK) according to the manufacturer's protocol. For the efficient inhibition of miRNA‐29, the cells were re‐transfected on day 3, photographed on day 4, and collected for RNA analysis or functional assays on day 5. Keratinocytes treated with miRNA‐29 inhibitors stayed longer in culture and were additionally collected on day 8 to check miRNA‐29 levels. All transfections were performed in biological and technical triplicates. A complete list of used miRNA mimics, anti‐miRNAs, cloning, and Taqman qRT‐PCR primers is provided in Table S4.

### miRNA‐29‐CLIP, followed by qPCR, QC, and NGS library preparation

100 μL of Prot G Dynabeads (Life Technologies) per 15‐cm dish was washed thrice with 1 mL of citrate–phosphate buffer (25 mm citric acid, 66 mm Na_2_HPO_4_, pH 5.0). The Ago2 antibody (50 μg per 100 μL of Prot G Dynabeads) was immobilized in a total volume of 500 μL of citrate–phosphate buffer by gentle rolling for 1 h at 4 °C. The beads were washed thrice with 1 mL of NP40 lysis buffer and blocked with 1 mL of NP40 lysis buffer containing BSA (10 μg·mL^−1^) for 0.5 h at 4 °C. After three washes with 1 mL of NP40 lysis buffer, the beads were resuspended in 50 μL of the lysis buffer.

20 μL magnetic streptavidin beads per 15 cm dish were prepared as per manufacturer's protocol. The washed beads were blocked in binding and washing buffer with salmon sperm DNA (100 μg·mL^−1^), BSA (100 μg·mL^−1^) and heparin (100 ng·mL^−1^) for 1 h at 4 °C on a rotator. The beads were washed five times with binding and washing buffer and resuspended in 50 μL of binding & washing buffer for pre‐equilibration.

After probe transfection, cells were washed once with 10 mL of PBS, placed on ice, and irradiated twice at 254 nm (CL‐1000 UltraviolettCrosslinker, UVP) with 100 mJ, followed by irradiation at 365 nm with 150 mJ. After irradiation, cells were scraped in 1.5 mL ice‐cold PBS, pelleted at 200 ***g*** at 4 °C for 5 min, and lysed in 1 mL of NP40 lysis buffer for 15 min on ice. The lysate was cleared at 14 000 ***g*** at 4 °C for 15 min and incubated with the Ago2 (clone 9A11; Ascenion, Munich, Germany) antibody‐coupled Prot G beads for 1 h at 4 °C with gentle rolling. The beads were washed five times with each 1 mL of IP wash buffer [50 mm HEPES, pH 7.5, 300 mm KCl, 0.05% NP40, 0.5 mm DTT, complete protease inhibitor (Roche, Welwyn, UK)]. After the last washing step, 200 μL digest buffer (100 mm Tris–HCl, pH 7.5, 150 mm NaCl, 12.5 mm EDTA) containing 100 μL proteinase K (recombinant PCR grade solution, Roche) was added to each sample and digested at 65 °C for 15 min. RNA from the proteinase K digest was isolated by chloroform/phenol/isoamyl alcohol (Life Technologies) with 20 μg·mL^−1^ Glycoblue (Life Technologies). After centrifugation for 10 min at 12 000 ***g***, the RNA was precipitated from the aqueous phase using 600 mL of 100% ethanol and 20 μL 3 m NaOAc at −20 °C overnight. Then, the samples were centrifuged at 12 000 ***g*** for 30 min at 4 °C to pellet the RNA, which was dissolved in 20 μL water.

For streptavidin affinity purification, Ago‐pull‐down samples were incubated with streptavidin beads for 30 min at 4 °C with gentle agitation. The beads were washed four times with binding and washing buffer and treated with DNAse (PCR grade, Roche) for 10 min at 37 °C. Beads were washed once with 1 mL of high‐salt buffer. The beads were shaken twice with 50 μL of a solution of 95% formamide/10 mm aqueous EDTA, pH 8.2, for 2 min at 65 °C. The solutions were combined, filled with 100 μL of water and 20 μL of 3 N aqueous sodium acetate, and purified by chloroform/phenol (Life Technologies) extractions. The aqueous phase was isolated, and RNA was precipitated with 1 mL of 100% EtOH at −20 °C overnight with the addition of 20 μg·mL^−1^ Glycoblue (Life Technologies). Then, the RNA pellet was washed with 70% EtOH, centrifuged at 14 000 ***g*** for 10 min at 4 °C, and dissolved in 20 μL of water.

Library was prepared using Illumina stranded Total RNA prep ligation with Ribo‐Zero plus following the manufacturer's protocol. Input RNA, RNA isolated from Ago2 IP (miRNA‐29‐CLIP probe, 409 or mock) and miRNA‐CLIP–purified RNA was submitted for Illumina sequencing. A small proportion of miRNA‐CLIP‐purified RNA was analyzed by qPCR to control for successful enrichment of a set of validated miRNA‐29 targets as follows. After reverse transcription (Multi‐Scribe Reverse Transcriptase Kit, Life Technologies) of the isolated RNA, enrichment of target genes as normalized to GAPDH was measured by qRT‐PCR (TaqMan Fast Advanced Master Mix, Thermo Fisher Scientific, Waltham, MA, USA) on a QuantStudio 12K Flex Real‐Time PCR system (Thermo Fisher Scientific) following the manufacturer's protocol. Quality and integrity of the RNA samples were assessed using a 4200 TapeStation (Agilent Technologies, Santa Clara, CA, USA) and then libraries generated using the Illumina^®^ Stranded mRNA Prep. Ligation kit (Illumina Centre, Cambridge, UK) according to the manufacturer's protocol. Briefly, total RNA (typically 0.025–1 μg) was used as input material from which polyadenylated mRNA was purified using poly‐T, oligo‐attached, magnetic beads. Next, the mRNA was fragmented under elevated temperature and then reverse transcribed into first strand cDNA using random hexamer primers and in the presence of Actinomycin D (thus improving strand specificity while mitigating spurious DNA‐dependent synthesis). Following removal of the template RNA, second strand cDNA was then synthesized to yield blunt‐ended, double‐stranded cDNA fragments. Strand specificity was maintained by the incorporation of deoxyuridine triphosphate (dUTP) in place of dTTP to quench the second strand during subsequent amplification. Following a single adenine (A) base addition, adapters with a corresponding, complementary thymine (T) overhang were ligated to the cDNA fragments. Pre‐index anchors were then ligated to the ends of the double‐stranded cDNA fragments to prepare them for dual indexing. A subsequent PCR amplification step was then used to add the index adapter sequences to create the final cDNA library. The adapter indices enabled the multiplexing of the libraries, which were pooled prior to cluster generation using a cBot instrument. The loaded flow cell was then paired‐end sequenced (76 + 76 cycles, plus indices) on an Illumina HiSeq4000 instrument. Finally, the output data was demultiplexed and BCL‐to‐Fastq conversion performed using illumina's bcl2fastq software, version 2.20.0.422.

Unmapped paired‐end sequences from an Illumina MiniSeq sequencer were output in BCL format and converted to FASTQ format using bcl2fastq v2.20.0.422 (Illumina Centre), during which adapter sequences are removed. Resulting reads were between 34 and 75 nucleotides in length. Unmapped paired‐reads of 74 bp were interrogated using a quality control pipeline consisting of fastqc v0.11.3 and fastq screen v0.13.0. The reads were trimmed to remove any adapter or poor‐quality sequence using trimmomatic v0.36 (Max Planck Institute of Molecular Plant Physiology, Golm, Germany; PMID: 24695404); reads were truncated at a sliding 4 bp window, starting 5′, with a mean quality < Q20, and removed if the final length was less than 36 bp. The filtered paired‐reads were mapped to the human reference sequence analysis set (hg38/GRCh38) from the UCSC browser, using star v2.5.3a (Illumina Centre). The genome index was created using the comprehensive Gencode v25 gene annotation (http://www.gencodegenes.org/). Normalization and differential expression analysis was performed using deseq2 v1.18.1 on r v3.4.0 (European Bioconductor Society e.V., Heidelberg, Germany). Normalization of paired‐read counts and calculation of regularized log_2_ fold change, without estimation of gene expression dispersion was performed using deseq2 v1.18.1 on r v3.4.0. Genes were annotated using information extracted using Biomart for Ensembl v85, which corresponds to Gencode v25. Unspliced sequence (UTR, exon, introns) was extracted using Biomart for Ensembl v85. The resulting FASTA file was scanned using a Perl script to identify the regular expression matches to seed motifs on forward and reverse strands.

### Functional analysis of NGS results

The miRNA‐29 targets captured by the probe were divided into strong, weak, and no seed binding based on their miRNAANDA scores and classified based on the target expression levels in HEK293T cells. The DAVID platform v6.8 functional annotation clustering tool was employed with UP_KEYWORDS, UP_SEQ_FEATURE, COG_ONTOLOGY, BBID, BIOCARTA, KEGG_PATHWAY, INTERPRO, PIR_SUPERFAMILY, SMART, GOTERM_BP_DIRECT, GOTERM_CC_DIRECT, and GOTERM_MF_DIRECT as clustering features. Clusters containing enriched terms were sorted by size and given the nomenclature based upon the terms included. Predicted target transcripts of miRNA‐29 were obtained from the micro‐TDS tool on the DIANA website. The predicted miRNA‐29 target transcripts with a *z*‐score above 0.3 were used for further comparison.

Ingenuity Pathway Analysis (IPA) was used to carry out a comparative analysis of fast adhering and slow adhering datasets. The color reflects the direction of change for the gene expression, with red indicating a high positive *z*‐score. The intensity of the colors indicates the prediction strength.

### qRT‐PCR and miRNA expression analysis

Total RNA was isolated using Trizol reagent (Invitrogen, Paisley, UK) and used for cDNA synthesis with random hexamer primers for measuring the primary transcript levels. TaqMan miRNA‐specific assays (Ambion, Paisley, UK) were used to measure miRNA‐29 levels and normalized to RNU (Thermo Fisher Scientific). For mRNAs, qRT‐PCR was performed with the SensiFast cDNA synthesis kit (Bioline, London, UK) using the manufacturer's protocol, followed by real‐time qPCR in 96‐ and 364‐well relative quantification protocols (from Applied Biosystems, Altrincham, UK and BioRad, Watford, UK, correspondingly) using *RPL27* as the reference gene.

### miRNA *in situ* hybridization combined with immunofluorescence

Based on previous work on miRNA *in situ* hybridization analysis with fluorescently labeled LNA probes, we developed a modified miRNA‐29 *in situ* hybridization protocol where the probes specifically hybridizing miRNA‐29a, miRNA‐29b, or a scrambled sequence of the same length are modified at the backbone and labeled with Cy2 [12, 23]. Briefly, cryopreserved mouse wound biopsies were sectioned (14 μm) and fixed with 4% paraformaldehyde (PFA). miRNA‐29a and scrambled sequences (48), synthesized and Cy2‐labeled by RiboTask, were hybridized at 63 °C (miRNA‐29a) or 55 °C (scrambled). Sections of the mouse hippocampus, which express high levels of miRNA‐29a, were used as a positive control, whereas skin sections from *miRNA29ab1* knockout mice, hybridized with the miRNA‐29a probe, as well as sections hybridized with the scrambled probe, served as negative controls to find the optimal temperature for hybridization and washing steps [12]. The antibody for K10 (DAKO, Agilent Technologies) and fibronectin (FN) were added at the last step of the wound section incubation and visualized using the secondary anti‐mouse Cy3‐ and anti‐rabbit Cy5‐labeled antibody (Thermo Fisher Scientific).

### Adhesion assay

Following the transfection of IFK with antisense‐miRNAs inhibitors or with sense miRNA mimics, the cells were seeded on fibronectin‐coated wells (8 μg·cm^−2^) and incubated for 5 min at 37 °C, 5% CO_2_. The cells that attached within the 5 min were considered fast‐attaching cells and the rest were slow‐attaching cells. After 5 min, the media was removed along with the slow‐attaching cells and fresh growth media was added. The cells were allowed to attach completely before adding PrestoBlue (Thermo Fisher Scientific) at a 1 : 10 dilution to determine the number of cells that are attached.

### ECM deposition assay

DF were treated with miRNA‐29 and control mimics or inhibitors and assessed for ECM proteins deposited over the course of 5 days using the BCA protein assay. As fibroblasts are fast‐dividing cells, the rate of dilution of the transfected oligonucleotides increases with the extended period of incubation. ECM deposition was therefore calculated by dividing the initial transfected oligo amount by the cell number measured using PrestoBlue.

### Conditioned media from DF

HDF cells were transfected with antisense miRNA‐29 oligonucleotides (inhibitors) or control inhibitors as previously described and conditioned medium (CM) was collected after transfection. CM with the secreted extracellular components were centrifuged at 2000 ***g*** for 5 min to remove cell debris and filter sterilized using 0.22 μm filter. The medium was lyophilized in Scanvac Coolsafe Pro55′ supported by a “vacuubrand RC 6 hybrid” rotary vane pump to obtain completely dried powder, which was then reconstituted using Dulbecco's PBS to the required concentration for adhesion assay with keratinocytes.

### SDS/PAGE and western blotting

Cells were harvested in 1× lysis buffer (Cell Signaling, Leiden, the Netherlands) with protease cocktail tablets (Roche) and PhosSTOP (Roche). Cells were sonicated and left to solubilize for 30 min on ice. Then, cell lysates were centrifuged at 10 000 ***g*** for 10 min at 4 °C to remove insoluble debris. Protein concentrations were measured using the BCA reagent (BioRad). Cell lysates were separated by using sodium dodecyl sulfate electrophoresis‐polyacrylamide gel electrophoresis (SDS/PAGE) and transferred to nitrocellulose membranes. After blocking for 1 h with Intercept blocking buffer (LI‐COR, Lincoln, NE, USA) diluted 1 : 2 with PBS, membranes were incubated with SPARC, FERMT2, or αSMA primary antibodies diluted in antibody solution (1 : 2 blocking buffer/PBS containing 0.2% tween). Tubulin or GAPDH was used as a loading control. Detection was carried out with fluorescent secondary antibodies IRDye^®^ 800CW Donkey anti‐Mouse IgG and IRDye^®^ 680CW Donkey anti‐rabbit IgG visualized by LI‐COR Odyssey CLx imaging system. Image Studio Analysis Software (LI‐COR Bioscience) was used for imaging quantification.

### Immunofluorescence

Cells were plated onto coverslips in 12‐well plates at a density of 60 000 cells per well. The next day, cells were transfected twice on subsequent days with 200 nm of anti‐miRNA‐29 (abc) or non‐specific antisense (nsa). Then, media was changed, and cells were allowed to grow for an additional 72 h, after which cells were rinsed with Dulbecco's PBS containing 0.1 mm CaCl_2_ and 1 mm MgCl_2_ (PBS/CM) and fixed with 2% paraformaldehyde in PBS/CM for 30 min at room temperature. After fixation, cells were washed three times with PBS/CM and permeabilized with IF buffer (PBS/CM, 0.1% Triton‐X100, 0.2% BSA) for 10 min. After quenching with 50 mm NH_4_Cl in PBS/CM for 10 min, cells were rinsed and incubated with anti‐pan collagen IV antibody or αSMA (clone MAB1420, R&D Systems, Abingdon, UK) at 4 °C overnight. Cells were washed with IF buffer and incubated with fluorescent secondary antibody Alexa 488 for 1 h at room temperature. After washing, nuclei were counterstained with DAPI, and cells were then mounted using a Prolong Gold antifade medium (P10144, Invitrogen). Images were visualized using a Zeiss Axio fluorescence microscope. Fluorescence intensity was quantified in at least 10 images per condition using an in‐house software.

### Enzyme‐linked immunosorbent assay (ELISA)

ELISA for human SPARC was performed using a ready‐to‐go sandwich assay kit with positive controls purchased from R&D Systems and following the manufacturer's guidelines. The standard curve was generated using recombinant SPARC, and unconditioned fibroblast medium was used as a negative control and background. Absorbance was measured in triplicates from the conditioned medium from two independent experiments using control inhibitors‐transfected fibroblasts and fibroblasts transfected with miRNA‐29 inhibitors.

### Statistical analysis

*n* = 3; *P < 0.05, ***P < 0.001, ****P < 0.0001, one‐way or two‐way ANOVA for all data applicable unless otherwise mentioned. All experiments were done in technical and biological triplicates unless mentioned otherwise.

## Results

### miRNA‐29 defines epidermal growth in mouse wounds and in human skin equivalents

**Fig. 1:**
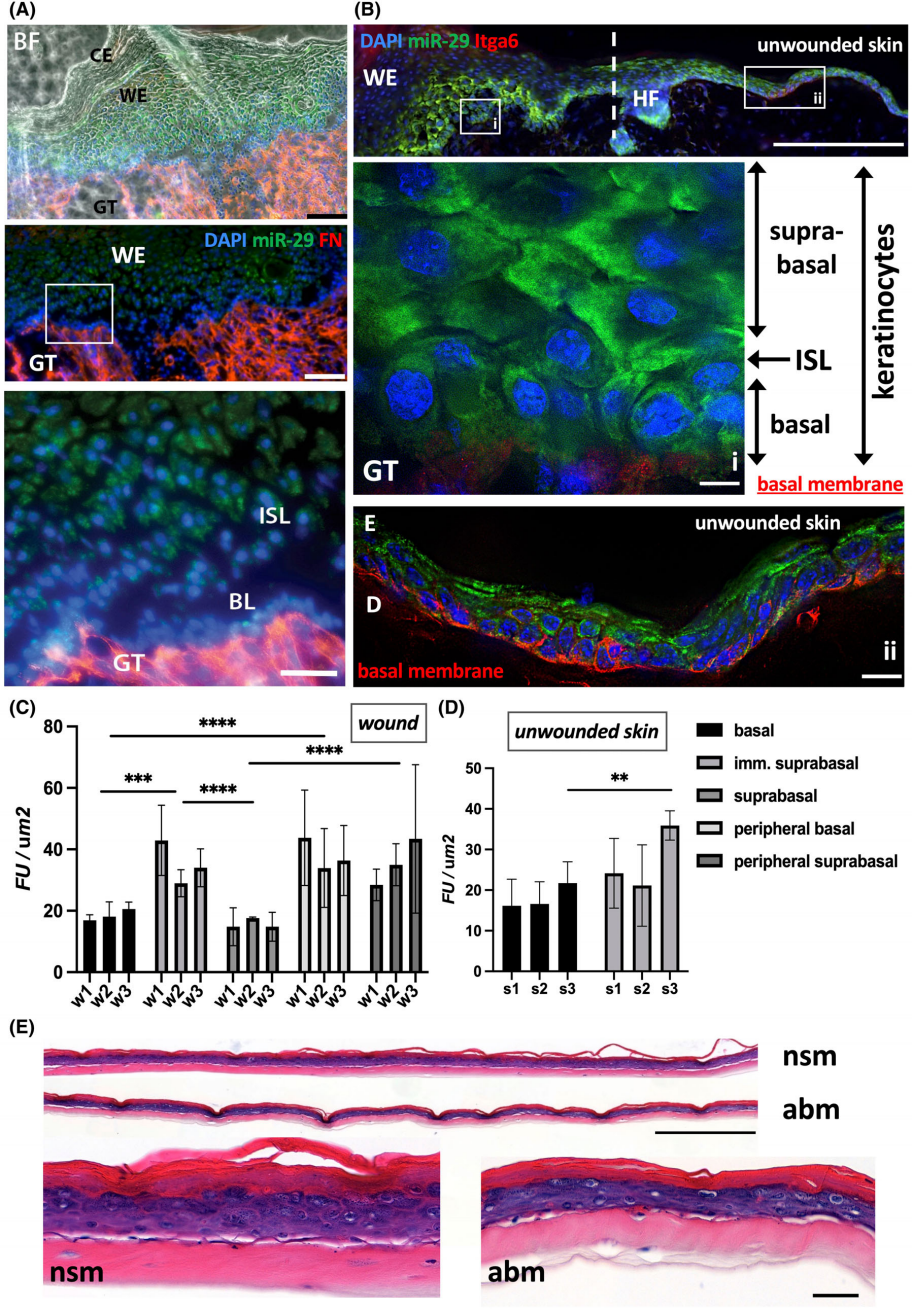
miRNA‐29 is differentially expressed in regenerating mouse epidermis and induces premature differentiation of keratinocytes in human skin equivalents. (A) Fully restored epidermis of a closed murine cutaneous wound at day 5 of healing shows miRNA‐29 localized to keratinocytes. miRNA‐29 is detected by *in situ* hybridization *(green)* co‐stained with fibronectin (FN, *red*), and DAPI (*blue*). Bright field (BF) shows newly formed cornified envelope (CE) and wound epidermis (WE). There is a distinct miRNA‐29 signal in WE, but the immediate suprabasal layer of the epidermis (ISL) and especially the basal layer (BL) attached to the granulation tissue (GT) shows scattered or no miRNA‐29. Scale bar = 100 and 50 μm. (B) Away from the wound bed, miRNA‐29 is expressed mainly in murine suprabasal epidermis transitioning from basal to differentiated suprabasal state, shown in the inset (i). miRNA‐29 signal in the inset (at the edge of the wound) appears more in basal cells than in the wound epidermis or in the unwounded. miRNA‐29 is *green*, nuclei are *blue*. Integrin alpha 6 (Itga6, *red*) shows adhesion to the basal membrane connecting epidermis to the underlying dermis. WE – wound epidermis, GT – granulation tissue, (ii) – unwounded skin, with intact E – epidermis, D – dermis. HF is a hair follicle, together with a punctate line marking the arbitrary border between wounded skin and the adjacent normal skin. Scale bars from top to bottom are 200, 10, and 20 μm. (C) Quantification of miRNA‐29 FISH signal in mouse wound epidermis (w) and in (D) unwounded skin (s). FU – fluorescence units. *N* = 3 (wounds or skin samples), *n* = 5 (areas of epidermis), **P < 0.01, ***P < 0.001, ****P < 0.0001, two‐way ANOVA followed by Tukey multiple comparison analysis. (E) Primary human keratinocytes were transfected with miRNA‐29 mimics (abm) or non‐specific sense oligo (nsm) and grown as SE for 7 days. Scale bar = 100 μm. Error bars indicate standard deviation of the mean.

Using *in situ* hybridization, we detected a strong signal for miRNA‐29a in all layers of mouse epidermis except the lowest single‐cell layer of keratinocytes attached to the basal lamina (Fig. 1A–D). Epidermis forming in the middle of the wound where basal keratinocytes migrate and adhere to fibronectin had relatively low expression of microRNA‐29, but the expression was activated in the immediate suprabasal layer (Fig. 1A,C). In light of the known function of miRNA‐29 in suppressing hyperadhesive desmosomes in mouse and human epidermis [14], we hypothesized that the mechanisms establishing adhesion have to be relieved of miRNA‐29‐mediated repression to help keratinocytes attach to the provisional matrix in wounds. These processes are crucial during wound closure as they regulate balanced migration and proliferation of keratinocytes, avoiding a chronic wound scenario. This balance may well be regulated by different levels of miRNA‐29 as it suppresses adhesion to allow both lateral and longitudinal migration of keratinocytes in the wound. We thus decided to investigate the mechanism of miRNA‐29‐mediated adhesion of newly forming epidermis as it was attaching to the underlying dermis. We observed more signal for miRNA‐29 in suprabasal epidermis in the peripheral part of the wound as well as in the unwounded epidermis (Fig. 1B,D). There was more miRNA‐29 at the basal layer of the epidermal tongue compared to the middle of the wound or unwounded skin (Fig. 1C), suggesting that a transient suppression of basal adhesion by miRNA‐29 is needed to allow keratinocyte migration. In line with our previous report that miRNA‐29 shows restricted localization to the suprabasal epidermis of normal human skin [12], we observed lower expression of miRNA‐29a in the basal layer of human *ex vivo* wounded skin explants compared to the suprabasal layer (Fig. S1A–C). Due to a limited size of human explant, we could not reliably compare the miRNA‐29 signal in the unwounded human skin to the miRNA‐29a levels in the wound. However, we found less suprabasal miRNA‐29a at the wound site compared to the epidermis at the periphery of the wound (Fig. S1B).

In human skin equivalents (SE), human keratinocytes transfected with the single‐stranded complementary oligonucleotide inhibitor of miRNA‐29a/b/c (abc) and seeded on dermal scaffolds of growth‐arrested fibroblasts and the ECM [24] formed thicker epidermis compared to the corresponding control (non‐specific antisense oligonucleotide, nsa) (Fig. S1D, quantified in Fig. S1H). Overexpression of microRNA‐29a/b mimic in keratinocytes growing in SE resulted in significantly thinner epidermis (Fig. 1E, quantified in Fig. S1H). This may be due to inhibited basal adhesion and premature differentiation of keratinocytes. Indeed, the suprabasal marker keratin K10 (K10) appeared in the basal layer of miRNA‐29‐transfected SE, while the SE transfected with control mimic and skin biopsy from a healthy human patient had little basal K10 (day 6) or K10‐negative (day 11) basal layer (Fig. S1E–G).

**Fig. 2:**
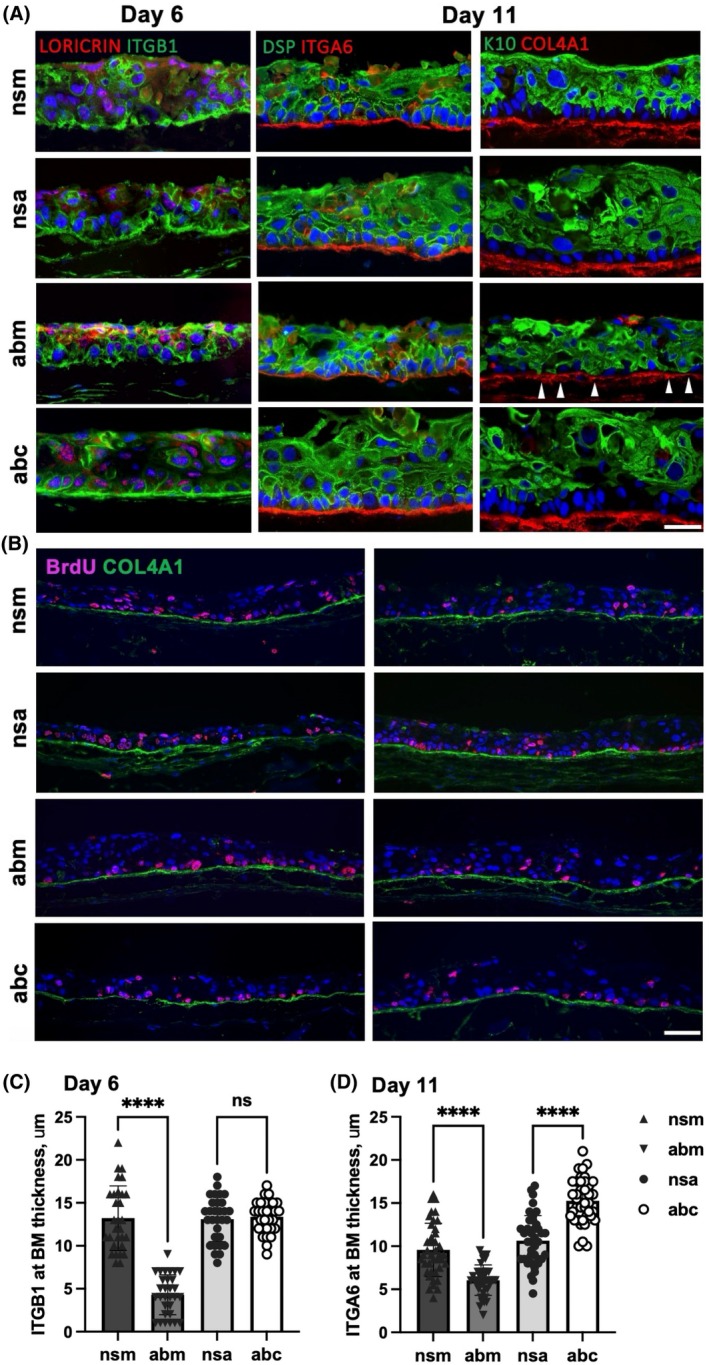
Inhibition of miRNA‐29 supports growth of keratinocytes in human skin equivalents. (A) Human skin equivalents (SE) grown in 3D cultures at air‐liquid interface for 6 and 11 days stained with the markers of basal adhesion (integrins ITGB1 and ITGA6), early (K10) and late differentiation (LORICRIN), and cell‐to‐cell adhesion (desmoplakin DSP). Non‐specific sense oligo (nsm) and non‐specific antisense (nsa) are controls for miRNA‐29 mimics (abm) or miRNA‐29 inhibitors (abc), respectively. White arrowheads indicate premature expression of K10 in the basal layer of keratinocytes at day 11 of SE when miRNA‐29 is overexpressed. Scale bar = 50 μm. (B) Bromodeoxyuridine (BrdU) staining of SE sections of keratinocytes transfected with non‐specific mimic (nsm), non‐specific inhibitor (nsa), miRNA‐29 mimic (abm), miRNA‐29 inhibitor (abc). Scale bar = 100 μm. The border between epidermis and the dermal scaffold is stained with collagen IV (COL4A1). (C) ITGB1 staining and (D) ITGA6 staining quantified at the BM surface. ****P < 0.0001, ns – not significant (*P* > 0.05), one‐way ANOVA followed by Tukey multiple comparison analysis. Error bars indicate standard deviation of the mean.

The epidermis was also thinner in SE made of immortalized human keratinocytes overexpressing miRNA‐29 (Fig. S2A–C). Interestingly, a major adhesion receptor and progenitor marker in the skin [25], integrin beta1 (ITGB1) was not detected at the basal lamina in these SEs treated with miRNA‐29 mimics (Fig. 2A, quantified in 2C) but was readily detectable in miRNA‐29 inhibitor‐treated SEs. Integrin alpha 6 (ITGA6), a ligand of laminin 332 responsible for cell‐to‐matrix adhesion of keratinocytes, was deposited close to the basal lamina marked by collagen IV (Fig. 2A, quantified in 2D). Visibly improved signal for ITGB1 after inhibition of miRNA‐29 was followed by significantly higher deposition of ITGA6 (Fig. 2A,D). These data suggest that inhibition of miRNA‐29 could improve the formation of basal lamina (BM). Importantly, the BrdU incorporation rate did not change in response to miRNA‐29 expression or its knockdown (Fig. 2B, quantified in Fig. S1I), confirming that miRNA‐29 does not regulate the proliferation of keratinocytes. Overexpression and inhibition of miRNA‐29 in SEs are quantified in Fig. S2D.

### miRNA‐29 targets are upregulated during fast adhesion of human keratinocytes

**Fig. 3:**
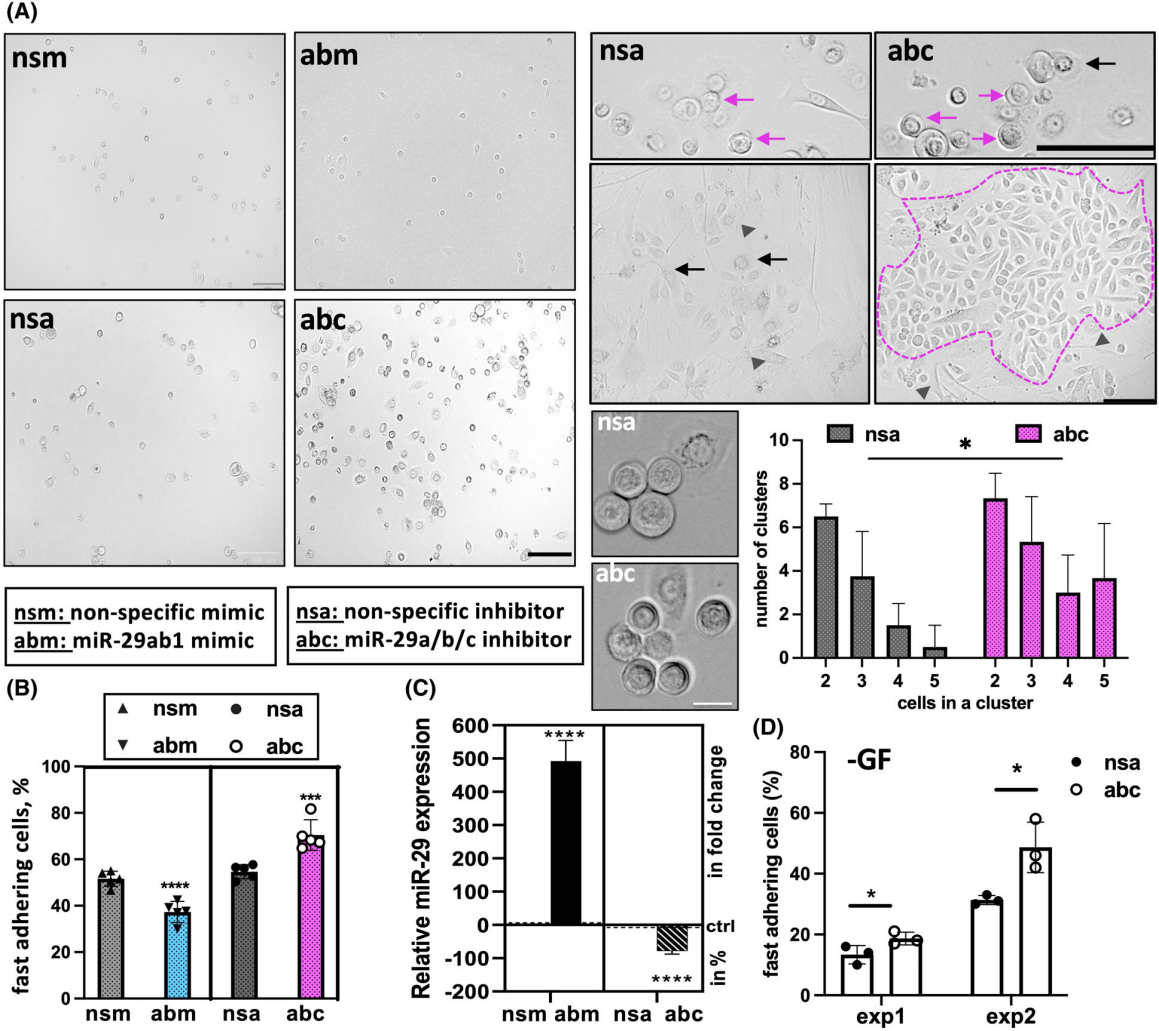
Loss of miRNA‐29 enhances basal keratinocyte adhesion. Human primary keratinocytes were transfected with 200 nm of miRNA‐29 inhibitors (abc) or non‐specific antisense (nsa) or 50 nm of miRNA‐29 mimics (abm) or non‐specific sense oligo (nsm) and the fast‐adhering cells were separated using fibronectin‐coated plates. (A) Representative images of the cells from each treatment. The arrows indicate clusters of more than two cells attached to the matrix, with purple arrows and a punctate line indicating clusters with a potential for clonogenicity. Arrowheads show keratinocytes that acquired a spindle‐like morphology with a low nuclear‐to‐cytoplasm ratio indicating lost potential to form a holoclone. Scale bar 100 μm (left four panels) and 50 μm (four panels on the right). Cell clusters were counted in each treatment (insets show 4 and 5‐clustered cases, scale bar 10 μm). *N* = 4, at least 50 cells counted per image, *P < 0.05, paired *t*‐test. (B) Number of fast adhering cells determined by PrestoBlue, ***P < 0.001, ****P < 0.0001, one‐way ANOVA. (C) Relative levels of miRNA‐29 were measured by TaqMan assays to confirm overexpression and inhibition in keratinocytes. Punctate line indicates miRNA‐29 levels in control samples. ***P < 0.0001, one‐way ANOVA. (D) Adhesion assay as in (A) was performed at two independent experiments (exp) in the absence of growth factors with two different batches of human keratinocytes (exp1 and exp2). Lower numbers of cells in exp1 as compared to exp2 are due to variability between batches of primary cells. Cells were transfected with miRNA‐29 inhibitor in triplicates, and total cell numbers were determined using PrestoBlue (*n* = 3). miRNA‐29 inhibition is quantified by TaqMan assays and shown in Fig. S2E. ***P < 0.001, two‐way ANOVA followed by Šídák's multiple comparison. Error bars indicate standard deviation of the mean.

It is well established that adhesion of primary human keratinocytes is essential for their proliferation and expansion [9]. To test if low levels of miRNA‐29 improve basal adhesion, we used a rapid (also known as fast) adhesion assay following overexpression or inhibition of miRNA‐29. High levels of miRNA‐29 abrogated fast adhesion of keratinocytes and stopped their further proliferation, whereas loss of miRNA‐29 significantly increased the number of keratinocytes rapidly attaching to fibronectin (Fig. 3A,B). The levels of miRNA‐29 inhibition are shown in Fig. 3C. Loss of miRNA‐29 allowed cells to form clusters, essential for keratinocyte growth and maintenance of regenerative potential (Fig. 3A). Importantly, enhanced adhesion following inhibition of miRNA‐29 was not dependent on the presence of growth factors in the cell culture medium (Fig. 3D and Fig. S2E), an observation important for potential therapeutic applications. Consistent with our previous data in human SE, loss of miRNA‐29 did not increase keratinocyte proliferation during the first 4 days in culture (Fig. S3A). When the miRNA‐29 inhibition experiment went beyond day 4 and until day 8, the control cells became cobble‐like and showed signs of spontaneous differentiation expected from primary keratinocytes growing in 2D culture for 7 days [12]. In contrast, cells that lost miRNA‐29 function continued proliferating until day 6, showing the morphology of expanding primary cells in 2D culture until day 8 (Fig. S3A,B). The levels of miRNA‐29 remained low following transfections with specific inhibitors (Fig. S3C). This suggested that the inhibition of miRNA‐29 supported the expansion of primary keratinocytes through improved cell‐to‐matrix adhesion.

It was thus very important to identify the mechanisms released by miRNA‐29 suppression in fast‐attaching cells. To do this, we knocked‐down miRNA‐29 in primary human keratinocytes with miRNA‐29 inhibitors and selected fast and slow adhering cells using fibronectin‐coated dishes. We then isolated and sequenced total RNA and compared the changes in gene expression between “fast” and “slow” keratinocytes with normal or depleted levels of miRNA‐29 as explained in Fig. S4A. In an initial exploratory data analysis, we performed principal component analysis (PCA) of the raw data which showed the differences between the transcriptome of these cell populations (Fig. S4B). Indeed, the EnrichR analysis confirmed the acquisition of many more biological pathways (BP), cellular compartments (CC), and molecular functions (MF) in fast adhering cells as a result of miRNA‐29 removal (compare Fig. S5A,B). Protein binding in response to stimulus in the cytoplasm most significantly contributed to fast adhesion upon inhibition of miRNA‐29 (Fig. S5C). This result suggested that inhibition of miRNA‐29 released the expression of molecules that activated adhesion though cascades of signaling, starting from the transmembrane receptor proteins interacting with the second messengers and engaging more collateral mechanisms. We could not identify any previously known adhesion mechanism strongly enriched in fast miRNA‐29 knockdown cells. It may be that miRNA‐29 repressed basal adhesion through multiple, previously uncharacterized nodes of regulation. These regulators could be both direct and indirect targets of miRNA‐29, forming a complex network. Thus, it was necessary to identify which mRNAs are bound and downregulated by miRNA‐29 directly.

### miRNA‐29 directly targets new master regulators of adhesion in human skin cells

**Fig. 4:**
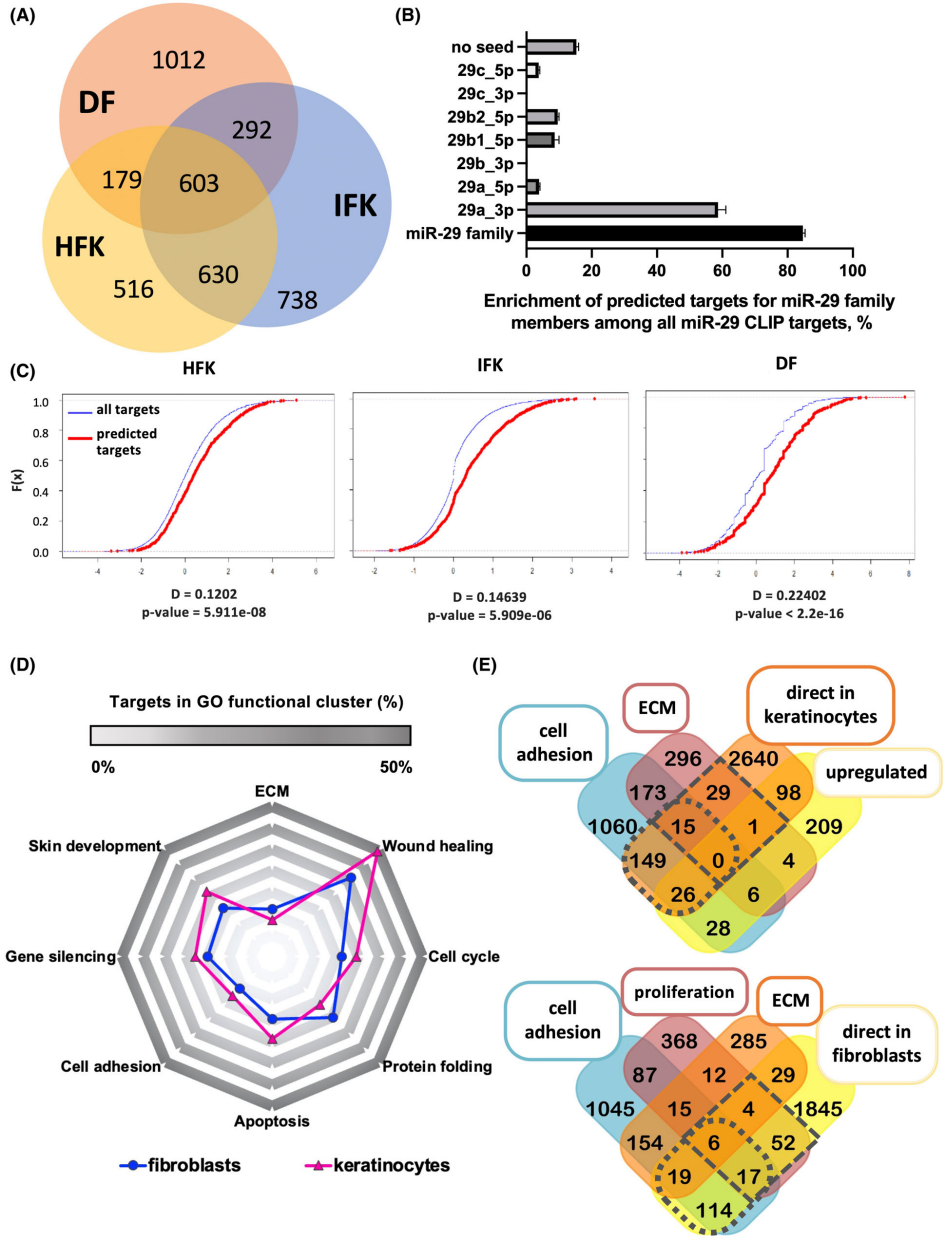
miRNA‐CLIP successfully identifies miRNA‐29 targetome in primary skin cells. (A) Venn diagram of an overlap between the miRNA‐29 targetomes (identified using miRNA‐CLIP) of skin cells. (B) Percentage of all predicted targets of the miRNA‐29 family members captured by the miRNA‐29 probe in miRNA‐29‐CLIPs. (C) Empirical Cumulative Distribution Function plotting the distribution of all miRNA‐29‐CLIP targets (blue) with predicted targets (red) against log2FC with the distance and *P* value for each of the cell types. This shows target enrichment achieved by the CLIP method vs. the target prediction. (D) Radar plot showing selected Gene Ontology (GO) terms represented in miRNA‐29 targetomes in fibroblasts and keratinocytes. (E) *Top*: Venn diagram showing the overlap of direct miRNA‐29‐CLIP targets with cell adhesion GO terms (punctate oval), ECM (square), and with the mRNAs upregulated by miRNA‐29 inhibition in keratinocytes. *Bottom*: the overlap of targets in GO term clusters for cell adhesion, proliferation, and ECM, with direct miRNA‐29‐CLIP targets in fibroblasts. Consistent with our data, proliferation function is absent in keratinocytes and the inhibition of miRNA‐29 is used to select upregulated targets of miRNA‐29 in fast adhering cells. Error bars indicate standard deviation of the mean.

To identify the mRNAs bound by miRNA‐29, we performed miRNA‐CLIP in three types of primary cells isolated from human skin. The first type was the human follicular keratinocytes (HFK), the epidermal stem cells isolated from the scalp. The second type was the human interfollicular keratinocytes (IFK) and the third type was the human dermal fibroblasts (DF), isolated from the neonatal skin. Biotinylated miRNA‐29 probe was transfected into HFKs, IFKs, and DFs, crosslinked to mRNA targets, and the AGO2 pull‐down was performed to isolate a functional RNA‐induced silencing complex (RISC). The RNA‐miRNA duplexes were then extracted from the AGO2‐RISC fraction and precipitated on streptavidin‐coated beads, isolating the RNAs bound by the miRNA‐29 probe. The resulting cDNA libraries thus contained the RNAs bound and targeted to the RISC by miRNA‐29. Before proceeding toward the sequencing of the libraries, quantitative PCRs were performed to ensure the enrichment of known and predicted miRNA‐29 targets in the probe fraction compared to the AGO2‐RISC (IP1) and in the input. A significant enrichment of miRNA‐29 targets was observed in all three cell types tested (Fig. S6A–C). Sequences of miRNA‐29‐CLIP libraries were analyzed based on the number of reads and their enrichment. The predicted targets of miRNA‐29 showed a significant enrichment in all three cell types (Fig. S6D). The HFK and IFK miRNA‐29 targetomes shared more than 64% (HFK) and 54% (IFK) of their targets with each other. 51% was shared between targetomes of keratinocytes and fibroblasts (Fig. 4A). This result shows different miRNA‐mRNA pairs even in the cells of the same or related origin and function (HFK and IFK). To determine the representation of miRNA‐29 targets in all RNAs captured by the probe, we used two prediction tools: (a) TargetScan, which predicts targets based on evolutionary conservation, and (b) microT‐CDS, which uses a machine‐learning approach to detect miRNA‐target interaction criteria from experimental data [26, 27, 28]. As expected, miRNA‐29a‐3p targets had the highest representation (~ 60%, Fig. 4B), correlating to the abundance of this miRNA‐29 compared to other family members, and most of the miRNA‐CLIP mRNAs were predicted targets of the miRNA‐29 family for all three cell types (Fig. 4C).

To compare miRNA‐29 targetomes in HFK, IFK, and DF, we performed cluster analyses of miRNA‐29 CLIP based on GO terms. When we chose the GO relevant to skin repair and barrier functions and accessed the percentage of miRNA‐29 targets in them, we found that miRNA‐29 targets cluster the most in the wound healing category for both keratinocytes and fibroblasts (Fig. 4D). 45 transcripts were direct targets of miRNA‐29 in keratinocytes and associated with ECM, and 190 mRNAs were direct targets of miRNA‐29 in keratinocytes and associated with cell adhesion (demarcated by the punctate shapes in Fig. 4E). The equivalent numbers of direct targets of miRNA‐29 in ECM and cell adhesion are shown for fibroblasts, including cell proliferation (Fig. 4E, lower panel). 58 mRNAs with functions in ECM were directly bound by miRNA‐29 in total, with 10 functioning also in proliferation, and 25 – in cell adhesion (Fig. 4E, lower panel). We did not find cell proliferation as a function regulated by miRNA‐29 in keratinocytes, consistent with the lack of change in BrdU incorporation in keratinocyte 3D cultures following miRNA‐29 knockdown or overexpression (Fig. 2B). Comparing the fast adhesion targets upregulated by miRNA‐29 knockdown (KD) to direct targets of miRNA‐29 from CLIP resulted in 26 miRNA‐29‐mRNAs with the function in adhesion (Fig. 4E). Interestingly, zero mRNAs belonged to the ECM‐related adhesion in keratinocytes, suggesting that miRNA‐29 targeted mRNAs endogenous to keratinocytes, which were not involved in the direct regulation of the ECM. The overlap of miRNA‐CLIP targets from fibroblasts with GO clusters showed that, in contrast to keratinocytes, miRNA‐29‐mediated adhesion in fibroblasts is connected to proliferation and deposition of the ECM (Fig. 4E).

**Fig. 5:**
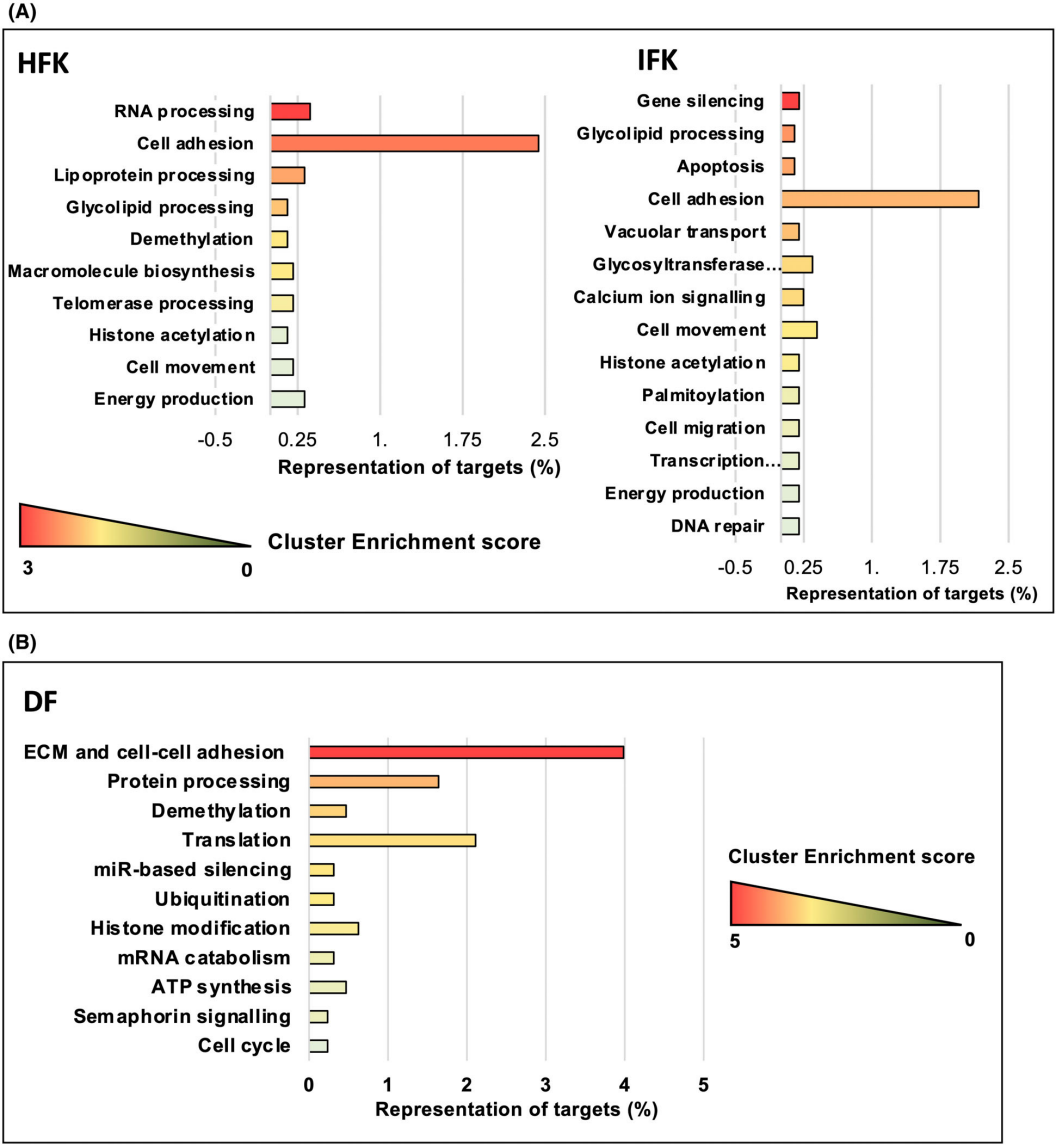
Functional analysis of miRNA‐29‐CLIP targetome in primary skin cells using DAVID. Functional analysis of the miRNA‐29‐CLIP targetome using the DAVID online tool for (A) human follicular keratinocytes (HFK) and interfollicular keratinocytes (IFK), and (B) dermal fibroblasts (DF). Proportion of miRNA‐29 targets represented in each of the clusters is on the *x* axis, and the color of the bars represents the cluster enrichment score.

In line with the data, cell adhesion was the most significant category in keratinocytes and fibroblasts when all miRNA‐29‐CLIP targets were analyzed by DAVID (Fig. 5). EnrichR showed mRNA clusters describing skin‐related functions and suggested that focal adhesion was more dependent on miRNA‐29 in follicular keratinocytes compared to the interfollicular cells (Fig. S7). miRNA‐29 was also directly involved in regulating the interaction with immune cells and lipid metabolism in keratinocytes and fibroblasts, with the IFK having the highest representation of lipid metabolism, and DF showing miRNA‐29 function in the cell cycle and DNA methylation (Fig. S7). Thus, many targets of miRNA‐29 appeared in multiple pathways during GO annotations, confirming our hypothesis about miRNA‐29 regulating cluster functions rather than individual adhesion pathways.

**Fig. 6:**
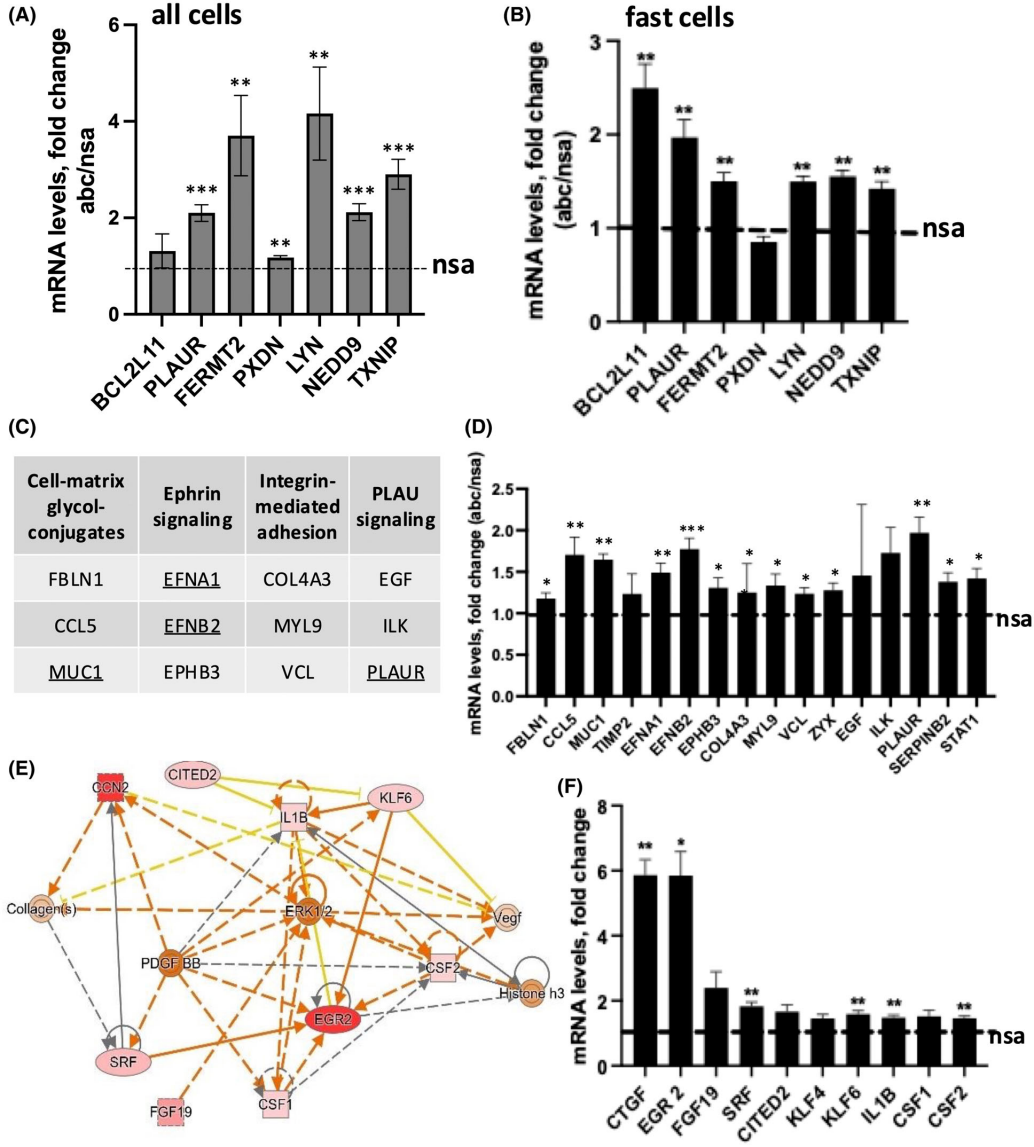
Identifying factors that contribute to enhanced adhesion in miRNA‐29 knockdown cells. (A) Fold change of miRNA‐29‐dependent regulators of adhesion in all keratinocytes and (B) fast adhering keratinocytes only. Cells were transfected with miRNA‐29 inhibitors (abc) versus control (nsa) and quantified using qPCR. Baseline expression of the nsa‐treated samples is set to 1 in (A) and (B); a two‐way ANOVA followed by Šídák's multiple comparison test resulted in *P* value * < 0.05, ** < 0.01, *** < 0.001. (C) mRNAs that represent four pathways related to epithelial cell adhesion were selected and changes in expression after miRNA‐29 inhibition were plotted in (D). The most significantly upregulated mRNAs are underlined in (C). (E) Ingenuity Pathway Analysis (IPA) generated map of functions from the fast keratinocytes (abc) versus control (nsa). The intensity of the colors indicates the prediction strength, with red indicating high positive *z*‐score. (F) Regulators of TGF‐ß/ß‐catenin pathway were selected and their fold change in abc‐ versus nsa‐treated fast keratinocytes were plotted (*N* = 3), unpaired *t*‐test: *P < 0.05, **P < 0.01, ***P < 0.001. Error bars indicate standard deviation of the mean.

To further test this, we manually searched for the functions of miRNA‐29 target mRNAs in each of the relevant pathways, choosing the mRNAs with a known role and measuring their expression following miRNA‐29 inhibition. We confirmed *PLAUR, FERMT2, LYN, NEDD9*, and *TXNIP* as direct targets of miRNA‐29 in the total and fast adhering populations of primary human keratinocytes (Fig. 6A,B). In addition, *BCL2L11* was specifically upregulated by miRNA‐29 inhibition in fast adhering keratinocytes (Fig. 6B).

Further analysis of the daughter categories of cell adhesion pathways (Table S1) identified miRNA‐29‐dependent regulators of early cell adhesion that could be grouped into four pathways that function in basal keratinocytes (Table S2 and Fig. 6C). *MUC1, EFNA1*, and *EFNB2* were significantly upregulated in the fast cells after inhibition of miRNA‐29 (Fig. 6D), suggesting the involvement of cell‐matrix glycol‐conjugates and ephrin signaling in fast adhesion. In addition, we analyzed miRNA‐29‐CLIP targets in TGF‐β and β‐catenin pathways (Fig. 6E). Ingenuity Pathway Analysis (IPA) identified more regulators of miRNA‐29‐controlled adhesion, namely, *CTGF, EGR2, SRF, KLF6, IL1B*, and *CSF2* (Fig. 6E,F). These results confirmed that miRNA‐29 controlled adhesion through pathways with distinct functions [29]. When upregulated, these pathways could enhance basal adhesion, each contributing only a part of the miRNA‐29‐controlled mechanism. To our knowledge, this is the first time that the regulators of adhesion are identified as directly regulated by a miRNA between human epidermal and dermal compartments, a cross talk that requires further investigation due to its high importance in the functionality and repair of human skin.

### miRNA‐29 regulates adhesion through ECM deposition and paracrine mechanisms

**Fig. 7:**
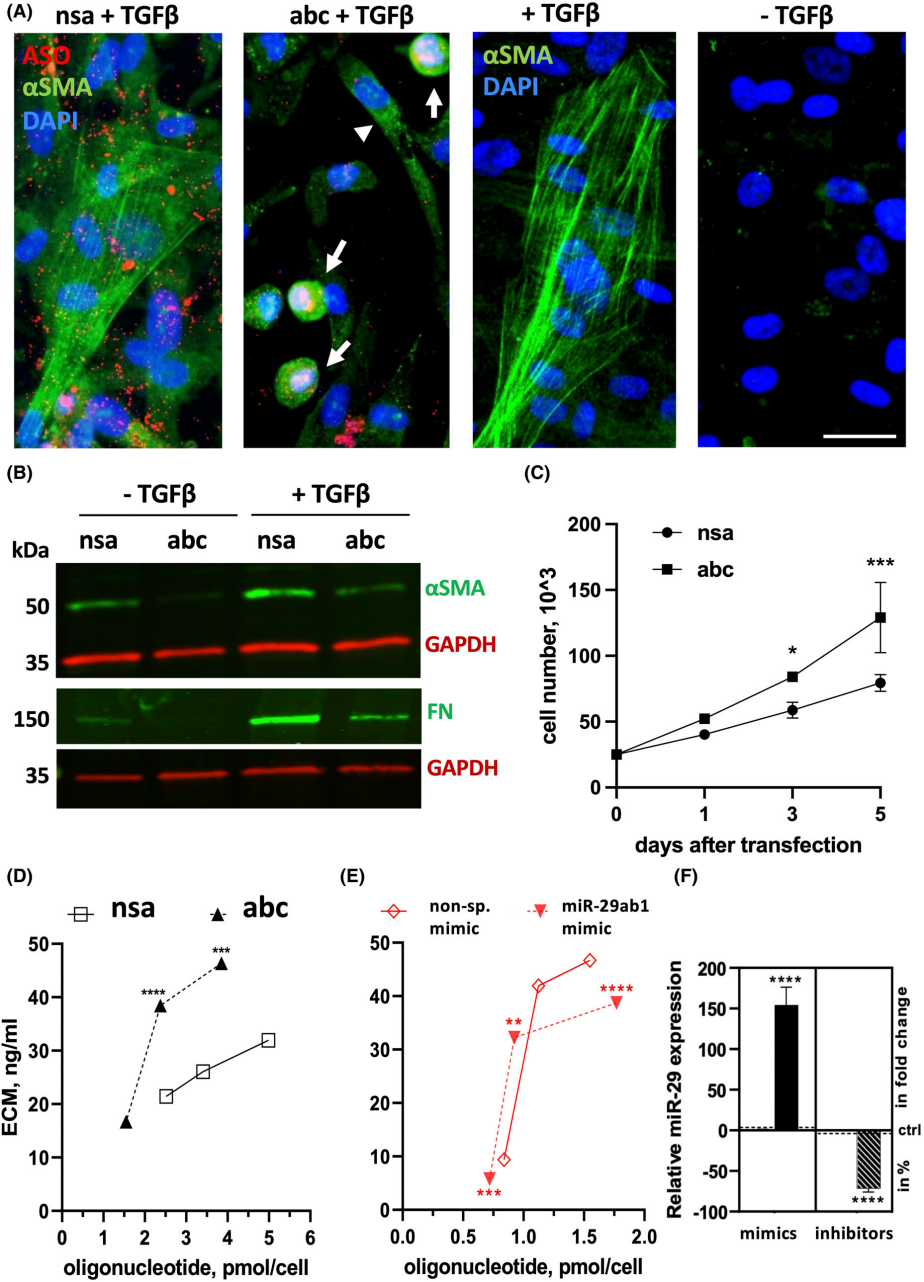
miRNA‐29 regulates proliferation and ECM deposition in dermal fibroblasts. (A) Expression of αSMA was assessed by immunofluorescence in dermal fibroblasts (DF) transfected with antisense miRNA‐29 (abc) or non‐specific (nsa) and treated with 1 ng·mL^−1^ of TGFβ1. Representative images are shown for nuclei staining with control (nsa) or miRNA‐29 inhibitors (abc) in red (antisense oligonucleotides, ASO), DAPI in blue, and αSMA in green. Arrows indicate localization of miRNA‐29 inhibitors inside the round‐shaped fibroblasts. Arrowheads point to a spindle‐shaped cell apparently not transfected with miRNA‐29 inhibitors. Untransfected cells are treated with TGFβ1 as a control for fibroblast into myofibroblast differentiation. Scale bar is 25 μm. (B) Expression of αSMA and fibronectin was assessed by the western blot in transfected fibroblasts after the treatment with TGF‐β1. GAPDH was used as a loading control. (C) Growth rate of DF was determined following miRNA‐29 inhibition (abc) compared to a non‐specific inhibitor (nsa). (D) DFs were transfected with miRNA‐29 inhibitors (abc) or non‐specific inhibitors (nsa) and (E) miRNA‐29ab mimic (abm) or non‐specific mimics (nsm) and the extracellular matrix (ECM) deposited by the cells was extracted by pepsin and quantified using BCA at 1‐, 3‐, and 5‐days post‐transfection. (F) Relative levels of miRNA‐29 were measured by TaqMan assays to confirm overexpression and the inhibition in DF. Punctate line indicates miRNA‐29 levels in nsm and in nsa control samples (both set to 1). Two‐way ANOVA followed by Šídák's multiple comparison test. *P < 0.05 or **P < 0.01 or ***P < 0.001 or ****P < 0.0001. Error bars indicate standard deviation of the mean.

Epidermal keratinocytes produce their own matrix as they attach on uncoated surfaces, whereas *in vivo* this process is coordinated with the underlying fibroblasts of the dermis. To further elucidate the dermal contribution to the miRNA‐29‐mediated regulation of adhesion, we used fluorescently labeled antisense oligos to inhibit miRNA‐29 in fibroblasts. Based on previously reported functions of miRNA‐29 in suppressing fibrosis, we expected to see an enhanced differentiation of fibroblasts to myofibroblasts. To our surprise, loss of miRNA‐29 did not lead to the appearance of myofibroblasts in culture and even suppressed fibroblast to myofibroblast conversion in response to TGFβ1 (Fig. 7A). Lower levels of smooth muscle actin (aSMA) in miRNA‐29 knockdown fibroblasts were confirmed by western blot (Fig. 7B). We also observed a decrease in fibronectin secretion by fibroblasts treated with miRNA‐29 inhibitors (Fig. 7B). At the same time, inhibition of miRNA‐29 significantly increased the number of fibroblasts at day 3–5 following transfection with inhibitors (Fig. 7C). It is worth noting that the miRNA‐29 inhibitors localized to rounded (as opposed to differentiated, aSMA‐positive) fibroblasts, suggesting that inhibition of miRNA‐29 supports their de‐differentiated state, allowing proliferation (Fig. 7A,C) and possibly, resulting in higher deposition of the ECM due to increased cell numbers.

Indeed, loss of miRNA‐29 resulted in significantly more ECM secreted by the fibroblasts (Fig. 7D) whereas addition miRNA‐29 suppressed ECM (Fig. 7E). While this result is overall consistent with the reported data on the miRNA‐29‐mediated fibrotic phenotype in skin [22], lung [30], kidney [31], and liver [32], it suggests that the mechanism of miRNA‐29‐regulated ECM deposition is not merely due to the de‐repression of ‘classic’ pro‐fibrotic targets. Rather, it may be the result of higher proliferative capacity of fibroblasts with low levels of miRNA‐29. Gain of miRNA‐29 function, however, may directly suppress the pro‐fibrotic targets, such as collagens I and III, resulting in less ECM (Fig. 7E). Levels of miRNA‐29 after overexpression and KD are shown in Fig. 7F.

**Fig. 8:**
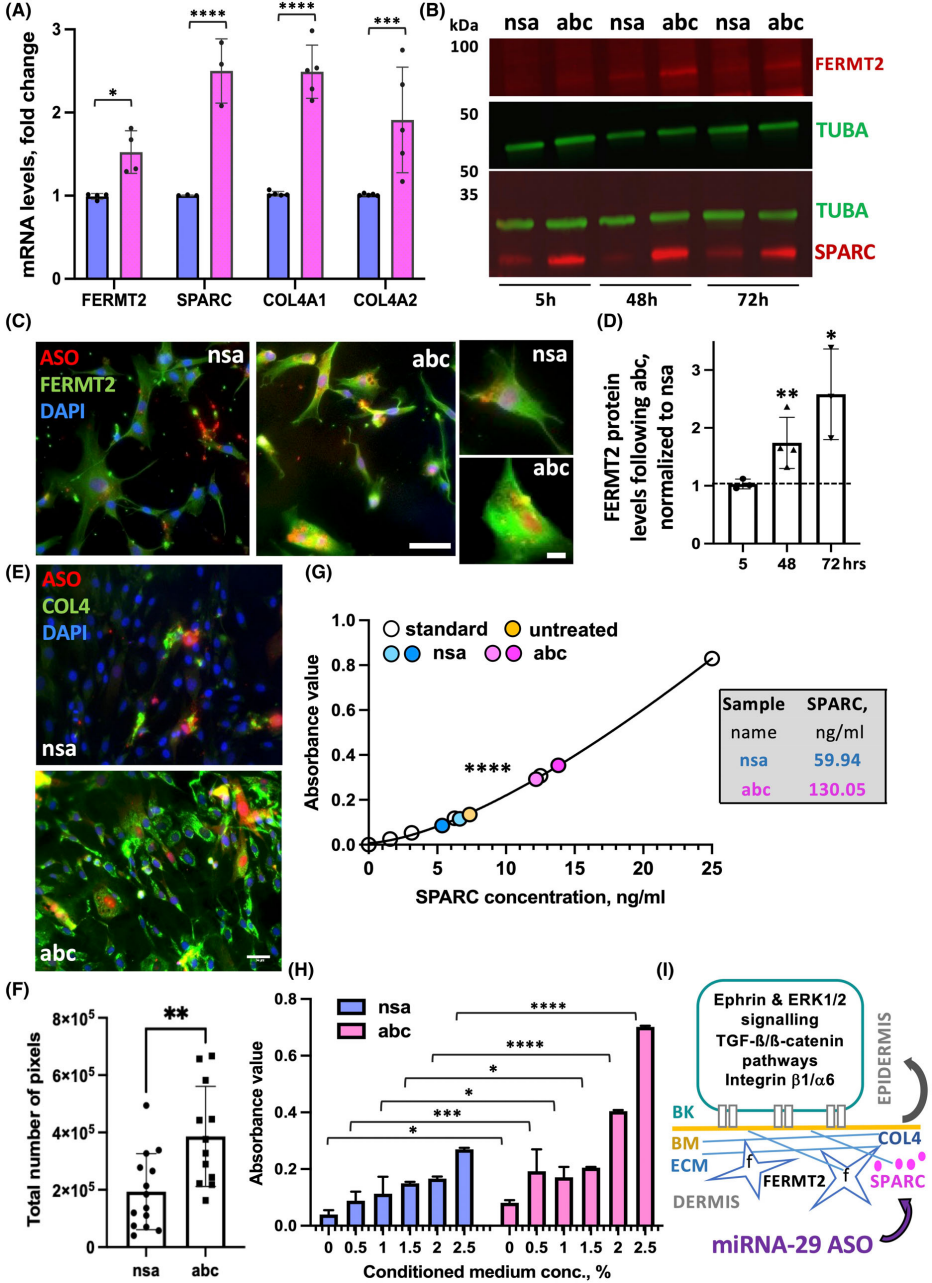
miRNA‐29 regulates ECM, proliferation, and adhesion through SPARC, FERMT2, and COL4. (A) Selected miRNA‐29 target genes represented in ECM and cell–cell adhesion cluster were confirmed by qPCR in human primary DF following inhibition of miRNA‐29 (magenta) vs. control (blue). (B) Expression of SPARC and FERMT2 was assessed by the western blot with tubulin as a loading control in DF transfected with miRNA‐29 inhibitors (abc) or control oligo (nsa). (C) Representative images for FERMT2 (green), miRNA‐29 inhibitors (abc) or control (nsa) antisense oligos in red (ASO) and nuclei (DAPI, blue). Scale bar = 25 μm. (D) Quantification of FERMT2 expression from western blots at 5, 48, and 72 h post‐transfection, *N* = 3, unpaired *t*‐test. *P < 0.05 or **P < 0.01. (E and F) Representative images (E) and quantification of collagen IV (F) using 10 images per condition. Unpaired *t*‐test, **P < 0.01. Scale bar = 35 μm. (G) Soluble SPARC was quantified by ELISA in the medium conditioned by miRNA‐29 inhibitors (abc) and control (nsa) inhibitors. Graph shows direct ELISA results where medium samples were diluted for precise quantification. The actual levels of SPARC are shown as numbers next to the graph. Both one‐way and two‐way ANOVA followed by Šídák's multiple comparison tests were performed, showing a highly significant increase in secreted SPARC, ****P < 0.0001. (H) Adhesion of keratinocytes was quantified on the increasing concentrations of conditioned media from miRNA‐29 inhibitors (abc) or control (nsa)‐treated fibroblasts. *N* = 3, unpaired *t*‐test, *P < 0.05 or **P < 0.01. The absorbance value coming from the PrestoBlue reagent corresponds to the number of alive adherent cells. (I) Interaction between proposed molecular players of enhanced keratinocyte adhesion regulated through miRNA‐29. BK – basal keratinocyte, BM – basal membrane, ECM – extracellular matrix, f – fibroblasts, and miRNA‐29 inhibitors – antisense oligonucleotides (ASO). Error bars indicate standard deviation of the mean.

To understand the mechanism by which inhibition of miRNA‐29 induces proliferation and ECM deposition in dermal fibroblasts, we went back to the miRNA‐CLIP targets involved in ECM deposition, proliferation, and adhesion (Figs 4E and 5B). Compared to the Venn diagram for keratinocytes, fibroblasts had more direct targets of miRNA‐29 represented in the ECM deposition category (1 vs. 10, correspondingly, Fig. 4E). We chose mRNAs that overlapped between ECM deposition and cell proliferation and were direct miRNA‐29 targets from the DF miRNA‐CLIP (Fig. 4E). We compared their expression in fibroblasts with suppressed miRNA‐29 and the control, confirming *FERMT2*, *SPARC*, *COL4A1*, and *COL4A2* as direct targets of miRNA‐29 (Fig. 8A). Inhibition of miRNA‐29 significantly increased the expression of SPARC protein as early as 5 h post‐transfection and remained elevated for 72 h, whereas FERMT2 protein responded to miRNA‐29 inhibition only after 24 h, probably reflecting differences in mRNA and protein turn‐around time (Fig. 8B–D). Consistently, fibroblasts expressing higher levels of FERMT2 had more rounded morphology compared to the star‐shaped control cells (Fig. 8C). The expression of *COL4A1* and *COLA4A2* increased by more than 2‐ and 1.75‐fold, respectively, confirming previous findings that these two mRNAs are direct targets of miRNA‐29 [33]. The increase in pan‐COL4 protein observed in the culture staining was visible and quantified (Fig. 8E,F), and confirmed regulation of collagen IV by miRNA‐29 in fibroblasts.

SPARC (secreted protein acidic and rich in cysteine) is an extracellular glycoprotein expressed in elevated levels in actively proliferating cells, with an indispensable function during wound healing [34]. SPARC has many post‐translational modifications resulting in various isoforms, with the shortest one (32 kDa, Fig. 8B) being secreted and involved in cell‐ECM interactions [35]. To validate the localization of increased levels of SPARC observed in fibroblasts with inhibited miRNA‐29 (Fig. 8B), we performed ELISA assays on the medium conditioned by miRNA‐29 inhibitor‐treated DFs vs. control inhibitors. As expected, SPARC was secreted by fibroblasts [36], and inhibition of miRNA‐29 significantly increased the concentration of SPARC in the medium compared to control (Fig. 8G).

Finally, we tested if these changes in the fibroblast secretome induced by miRNA‐29 inhibition can regulate keratinocyte adhesion via paracrine mechanisms. We collected the medium produced by DFs, which were transfected with miRNA‐29 inhibitors or control oligos. The conditioned medium was concentrated by lyophilization and added at increasing concentrations to untransfected keratinocytes as a substrate for the adhesion assay. We found that the number of rapidly adhering keratinocytes increased in the medium produced by miRNA‐29 inhibitor‐treated DFs compared to the control inhibitor conditioned medium (Fig. 8H). This result demonstrates that the inhibition of miRNA‐29 can also induce adhesion of basal keratinocytes through paracrine signaling from the underlying dermal fibroblasts (Fig. 8I).

Important to the mechanism of miRNA‐29‐mediated regulation, *FERMT2*, *COL4A1*, *COL4A2*, and *SPARC* (Fig. 8) showed high miTG score indicating strong binding of the target 3′UTRs to the miRNA‐29 ‘seed’ (Fig. S8). Thus, we report direct targets of miRNA‐29 that are endogenous to dermal fibroblasts and regulate proliferation, ECM deposition, and adhesion through signaling and paracrine mechanisms. We demonstrate that miRNA‐29 regulates adhesion in the skin by direct binding and regulation of mRNAs encoding proteins from many signaling pathways, including ephrin, MAPK/ERK, and TGFb/b‐catenin. We show that inhibition of miRNA‐29 increases basal adhesion of keratinocytes, supporting growth of the human epidermis *ex vivo*, and correlating to low levels of miRNA‐29 in basal epidermis in mouse wounds *in vivo*. These findings identify miRNA‐29 as a target for inhibitor‐mediated therapy, which has a potential to increase the regenerative capacity of epidermis and dermis during skin repair.

## Discussion

### miRNA‐CLIP identifies direct, cell‐specific, and functional targets of miRNA‐29

In this study, we aimed to investigate new functions of miRNA‐29 in the skin during its normal growth and regeneration. We chose human skin as a model to unveil the cell‐specific miRNA‐29 targetome in primary keratinocytes and fibroblasts, two major cell types of the skin, during their growth and differentiation in 2D and 3D models *ex vivo*. While we detected miRNA‐29 in human wounds *in situ*, an extremely limited supply of human skin biopsies and commonly used crosslinking methods of fixation at temperatures above 0 °C prevented us from getting results on miRNA‐mRNA pairs from wounds. It is difficult to expect an extended supply of human wound samples as their collection creates a larger wound. In the future, to achieve high‐quality analysis of miRNAs *in situ*, we recommend cryopreservation of biopsies with adjacent unwounded skin wherever possible.

A common approach identifies expression levels of target mRNAs after partial removal or an overexpression of a miRNA, followed by a target prediction through the “seed” sequence matching of the 3′UTR. Similarly, a pull‐down of the miRNA‐29 mimic labeled with biotin alone results in a capture of complementary RNA sequences outside of AGO2‐RISC, whereas the RNA immunoprecipitation (RIP) crosslinking precipitation (CLIP) of Ago2‐RISC does not reveal which miRNAs interacted with each of the isolated binding sites. Our approach, however, used low concentrations of the miRNA stem‐loop precursor, covalently linked to biotin and psoralen in nucleotide positions, which did not change the processing of the stem‐loop into the functional miRNA but allowed the tandem pull‐down of AGO2‐biotin complexes of miRNA‐29 and mRNAs, crosslinked near the ‘seed’ sequence [3].

### miRNA‐29 inhibition confers fast adhesion through multiple pathways

Cell adhesion plays an important role in skin regeneration and wound healing. Upon excisional wounding, the keratinocytes migrate over the fibronectin (FN) in the wound bed matrices for wound closure [37]. Also, FN reorganization in the basal membrane influences focal adhesion, which is essential in cell attachment and survival [38]. miRNA‐29 function at the edge of the wound may involve a transient regulation of basal adhesion where strong (i.e., fast) adhesion must be temporarily suppressed to allow keratinocyte migration toward the center of the wound.

Expression of ITGB1 and ITGA6 proteins was regulated by miRNA‐29 (Fig. 2A). ITGB1 and ITGA6 are co‐expressed in the basal layer of human skin [39] and SEs [40] and can heterodimerize to form integrin α6β1 in various tissues [41]. Single‐cell RNA sequencing identified ITGB1 and ITGA6 among the markers of epidermal stem cells and other undifferentiated progenitors in human skin [39]. In human SE, *ITGB1* and *ITGA6* showed high‐probability interactions with *LAMC2* during the EMT‐like gene expression signature driven by EGF signaling [40]. Considering this, our results suggest that miRNA‐29 mimics and inhibitors may be used to regulate the EGF‐mediated EMT in SE.

Adhesion molecules are proteins on the cell surface involved in the interactions between the cell surface and the ECM. Adhesion molecules include cadherins (subgroups E, N, P, M), integrins, selectins, and the immunoglobulin gene family. By RNA seq, we detected increased expression of cell adhesion molecules such as ICAM‐1/5, Nectin 2/4, CEACAM19, and L1CAM in the fast population following miRNA‐29 depletion (Table S3). All these molecules have been associated with increased cell adhesion and proliferation [42, 43].

### The regulation of extracellular matrix by miRNA‐29 is more complex and reversible than previously thought

Increased ECM deposition by fibroblasts is often associated with myofibroblast activation and TFGβ1 signaling. Many studies have reported the effect of TGFβ1 on the endogenous expression of miRNA‐29, but the effect of miRNA‐29 inhibition on TGFβ1 in primary dermal fibroblasts had not been reported [44]. Initial ECM assembly of latent TGFβ1 binding protein is strongly dependent on the presence of FN [37, 45]. Our results indicate that the miRNA‐29 family can regulate both TGFβ1‐dependent and ‐independent pathways.

From the direct targets of miRNA‐29 from the DF miRNA‐29‐CLIP, four targets involved in ECM deposition, cell adhesion, and proliferation specifically stood out. FERMT2, SPARC, and COL4A1/2 are released into the extracellular space and are associated with collagen fibril organization, contributing to ECM homeostasis [46]. FERMT2 activates integrins leading to FN binding and adhesion and subsequently assembles an essential signaling node at newly formed adhesion sites in a talin‐independent manner [47]. This directly contributes to the collagen IV network‐dependent FN and laminin assembly. SPARC binds a subset of ECM proteins, regulating ECM assembly through collagen interaction with the cell surface, and can be suppressed by miRNA‐29 [46, 48]. Our results identified endogenous targets of miRNA‐29 in fibroblasts and suggested that miRNA‐29 inhibition may facilitate *de novo* collagen synthesis and ECM assembly.

Taken together, our results revealed the direct targetome and functions of miRNA‐29 in three types of cells isolated from human skin. We uncovered new mechanisms of miRNA‐29‐regulated cell adhesion and deposition of the ECM and showed that these can be significantly enhanced by the inhibition of miRNA‐29. Our results demonstrate that changes in a single miRNA can influence different pathways in a cell‐type‐specific manner. When studied in the context of the organ, however, these pathways remarkably converge to regulate the most essential functions, such as restoration or the barrier formation in the skin. Since miRNAs can be up‐ or downregulated by short oligonucleotides, our findings open opportunities to regulate these functions by new therapeutic interventions.

## Author contributions

SK conceived, initiated, and supervised the project; LT, RS‐A, and CK designed and performed experiments; YW, FH, and JH designed the probe, modified the inhibitors, and assisted in the initial miRNA‐CLIP experiments; PR and LT analyzed the RNA sequencing data; H‐JS, IM, and PB developed, assisted, and analyzed 3D co‐cultures of human skin equivalents; LT, RS‐A, CK, and SK wrote and revised the manuscript. All authors approved the final manuscript.

## Peer review

The peer review history for this article is available at https://www.webofscience.com/api/gateway/wos/peer‐review/10.1002/1873‐3468.70051.

## Supporting information


**Fig. S1.** Gain of miRNA‐29 function results in differentiation of human keratinocytes but does not affect their proliferation.
**Fig. S2.** miRNA‐29 regulates growth of human SE.
**Fig. S3.** Inhibition of miRNA‐29 supports keratinocyte growth.
**Fig. S4.** Discovering miRNA‐29‐dependent and independent mechanisms of enhanced keratinocyte adhesion.
**Fig. S5.** miRNA‐29 regulates basal cell adhesion.
**Fig. S6.** Validation of miRNA‐29‐CLIP in primary skin cells.
**Fig. S7.** Functional analysis of miRNA‐29‐CLIP targetome in primary skin cells.
**Fig. S8.** Analysis of the ‘seed’ sequence match in miRNA‐29 targets 3′UTRs using DIANA tool.
**Table S1.** Cell adhesion pathways directly or indirectly regulated by miRNA‐29.
**Table S2.** Cell adhesion pathways directly or indirectly regulated by miRNA‐29 further analyzed from Table S1.
**Table S3.** Cell Adhesion Molecules.
**Table S4.** Oligonucleotide sequences used in analyses.
**Data S1.** Original Western blots show full size original blots scanned as described in the methods.

## Data Availability

The NGS data that support the findings of this study are available upon request as it contains very large datasets that are possible to interpret and further use when explained. The authors are happy to be contacted regarding miRNA‐CLIP and RNA seq analyses at any working time. All other extended data are also available from the University of Manchester repositories upon direct request made to the corresponding author.
