# Supplementary material for: miRNA‐29 regulates epidermal and mesenchymal functions in skin repair
Source: FEBS Lett. 2025 Apr 25;599(12):1795–817. doi: 10.1002/1873-3468.70051 (PMC12183617; doi:10.1002/1873-3468.70051)

Thiagarajan et al. Fig. S1

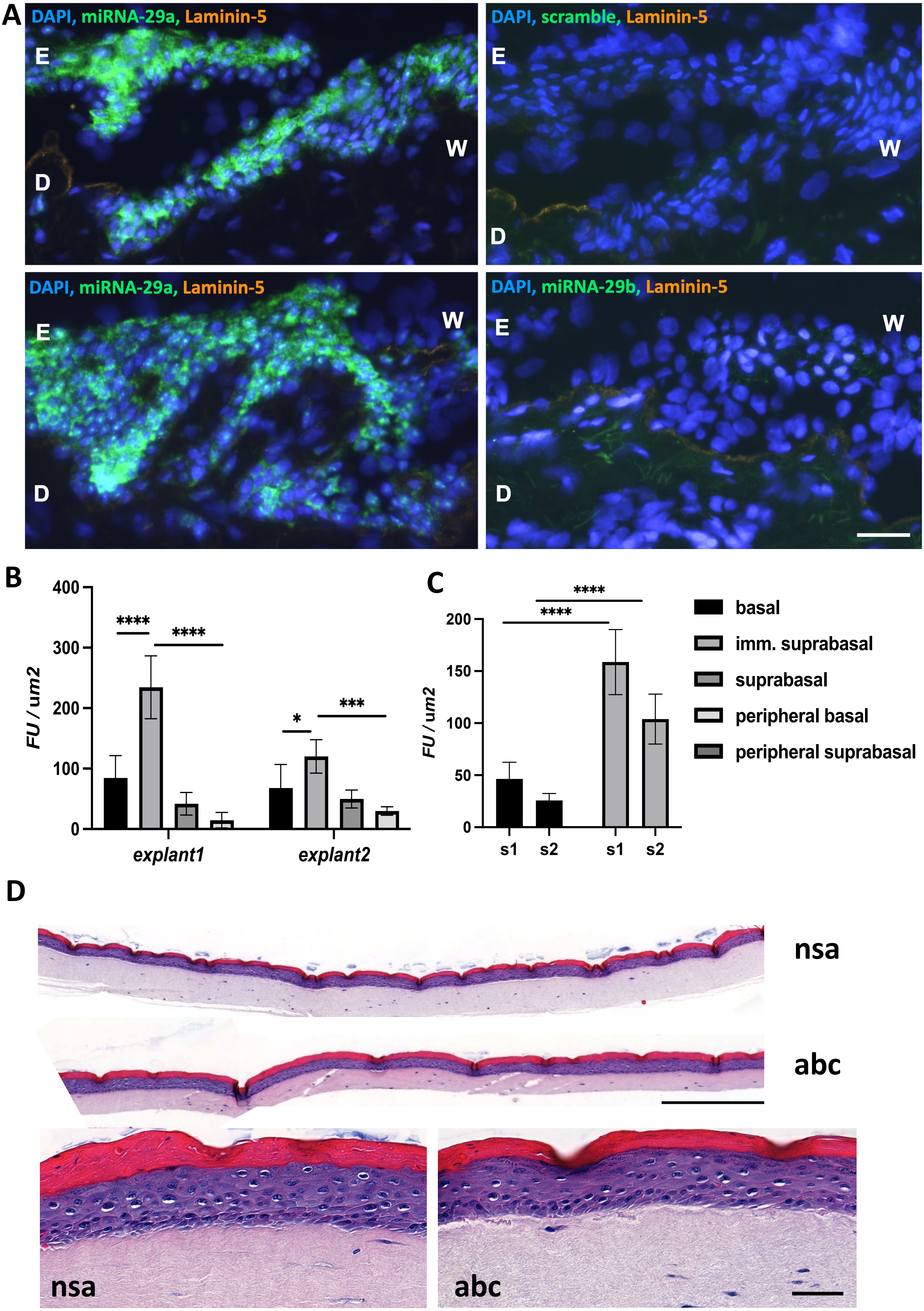

Thiagarajan et al. Fig. S1 (cont.)

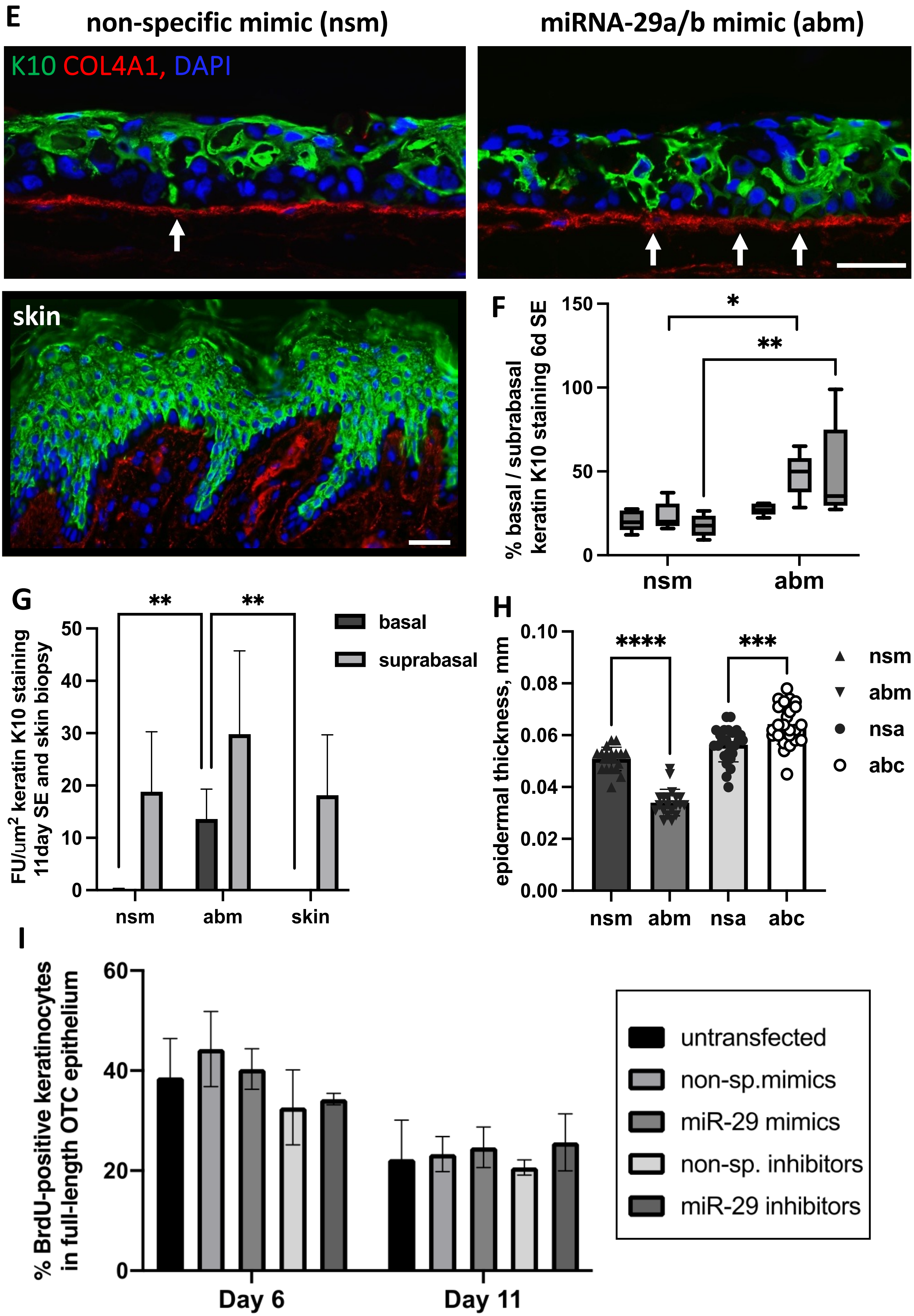

Thiagarajan et al. Fig. S2

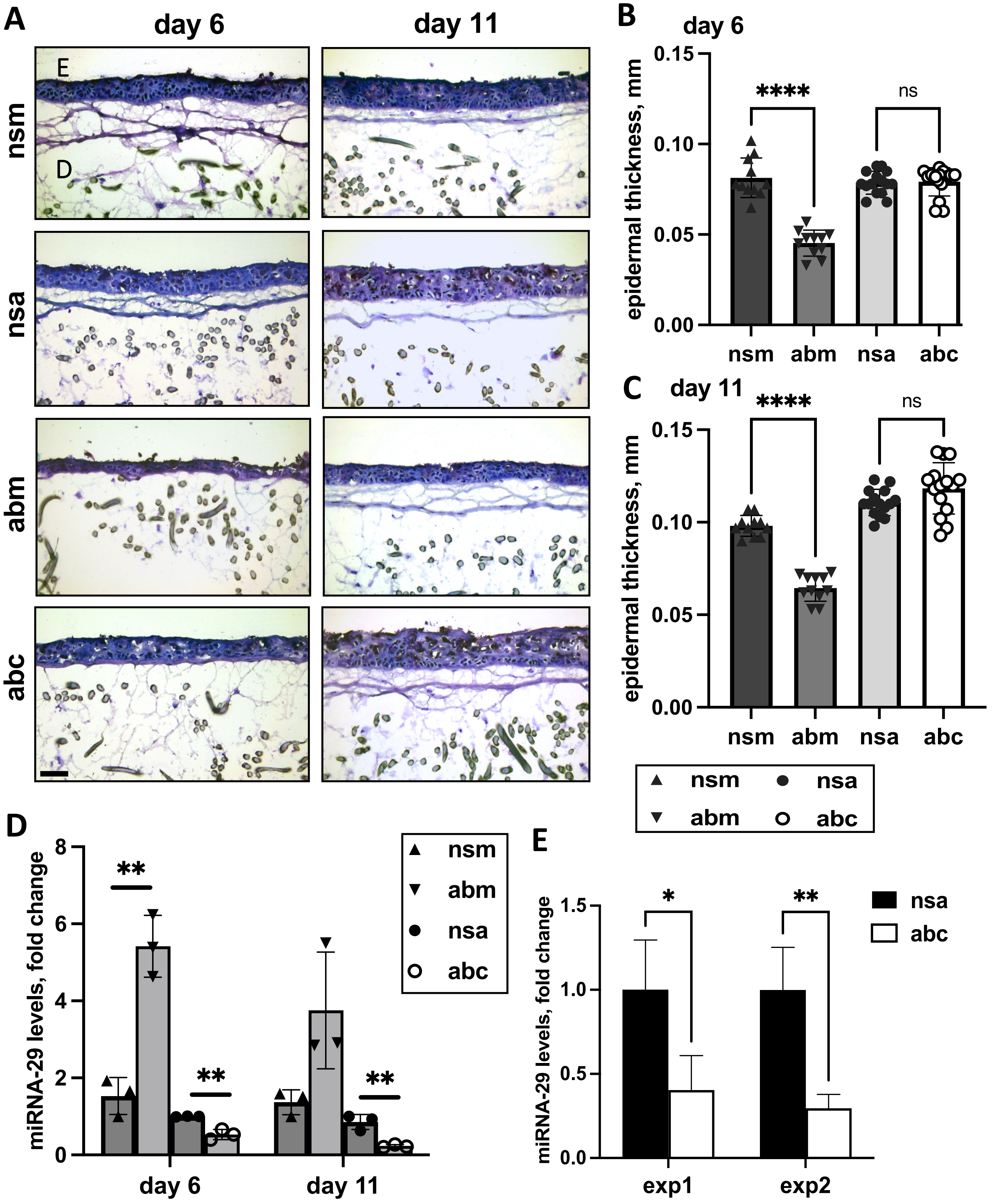

Thiagarajan et al. Fig. S3

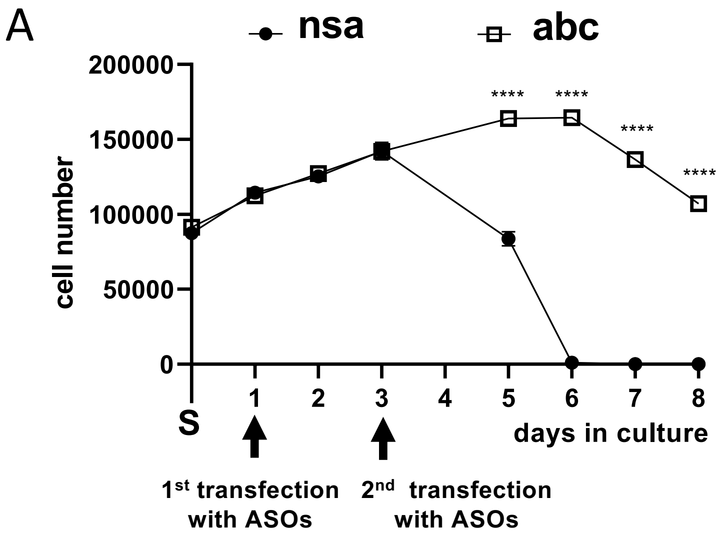

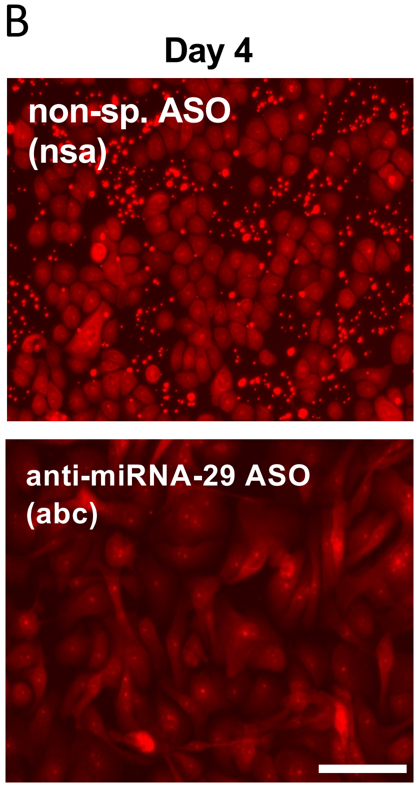

S: Start of the experiment, cells are plated at the same density
Day 1: cells are transfected with ASOs (1st)
Day 2: cells allowed to recover and continue to grow
Day 3: cells are transfected with ASOs (2nd)
Day 4: cells growth and transfection assessed in bright field and red (594nm) field, correspondingly
Day 5-8: cells transfected with non-specific ASOs stop growing, change morphology, and do not uptake the transfection particles (bright red dots outside of cells). Unlike the control nsa cells, the miRNA-29-ASO-transfected cells (abc) continue to grow until day 6 and appear normal until the end of the experiment (Day 8).

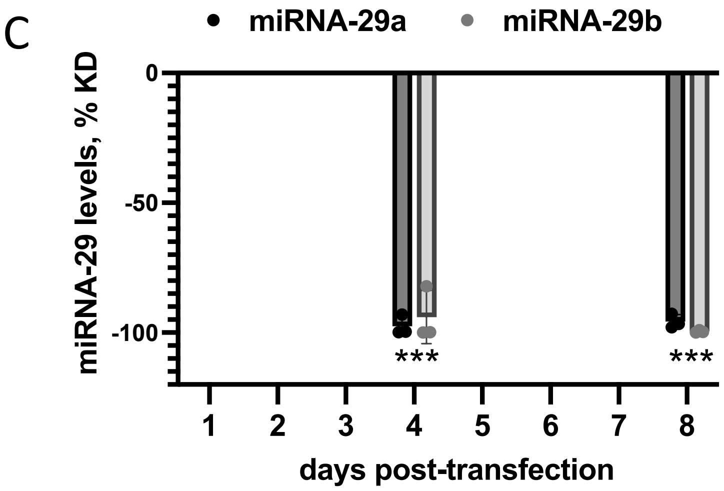

	% KD	P Value
miRNA-29a		
**nsa vs. abc**	**97.51**	0.0007
miRNA-29b		
**nsa vs. abc**	**94.00**	0.0009
miRNA-29a		
**nsa vs. abc**	**95.76**	0.0008
miRNA-29b		
**nsa vs. abc**	**99.55**	0.0006

Thiagarajan et al. Fig. S4

A

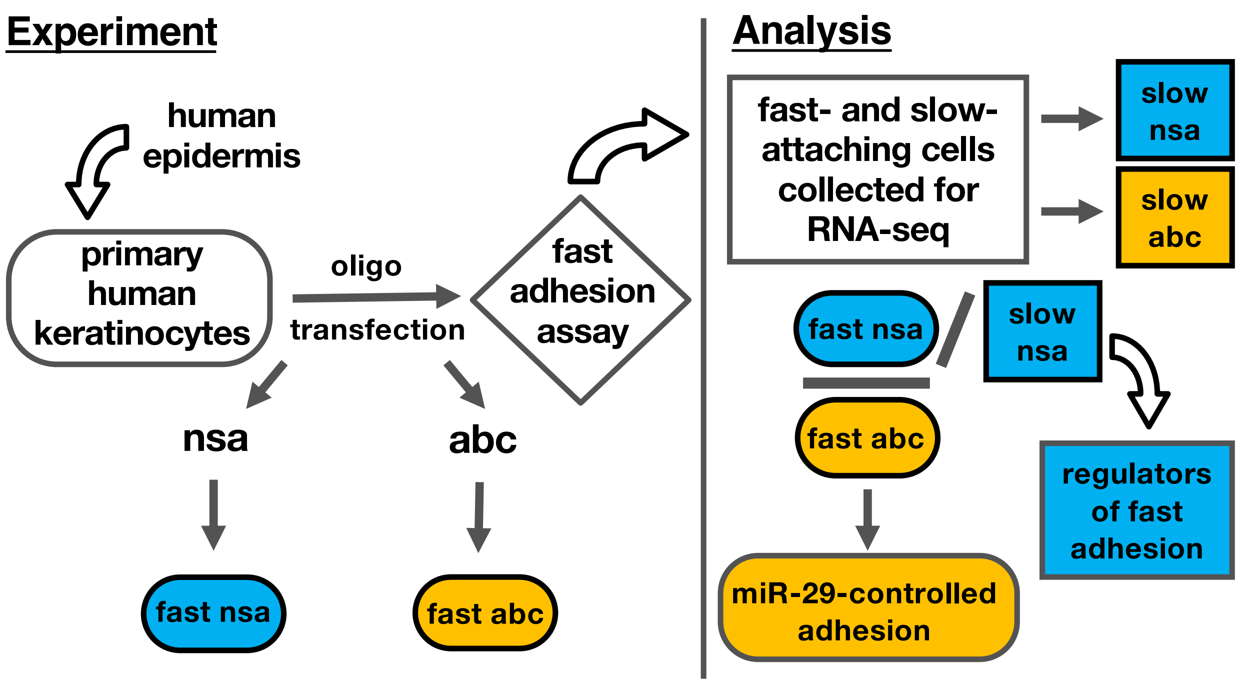

B

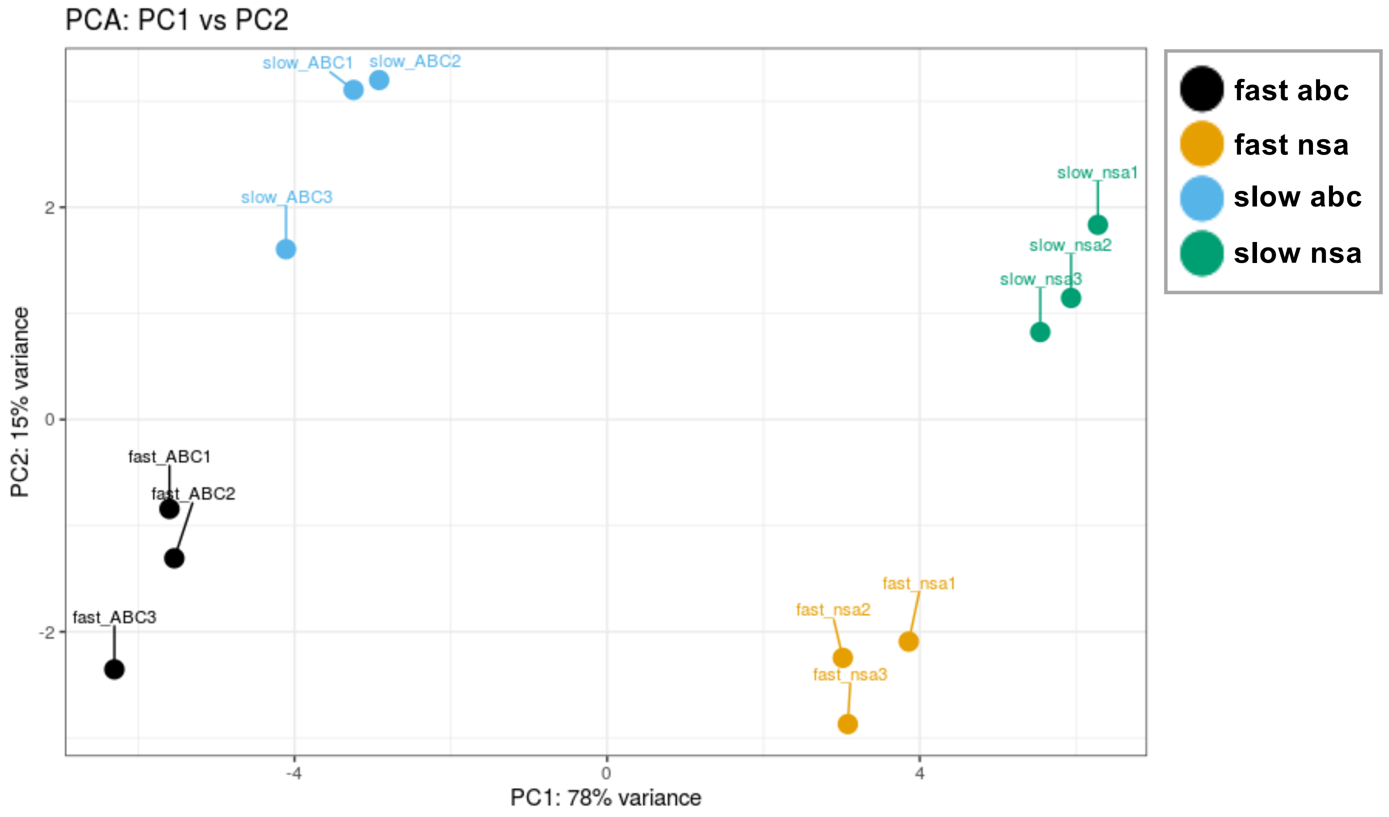

Thiagarajan et al. Fig. S5

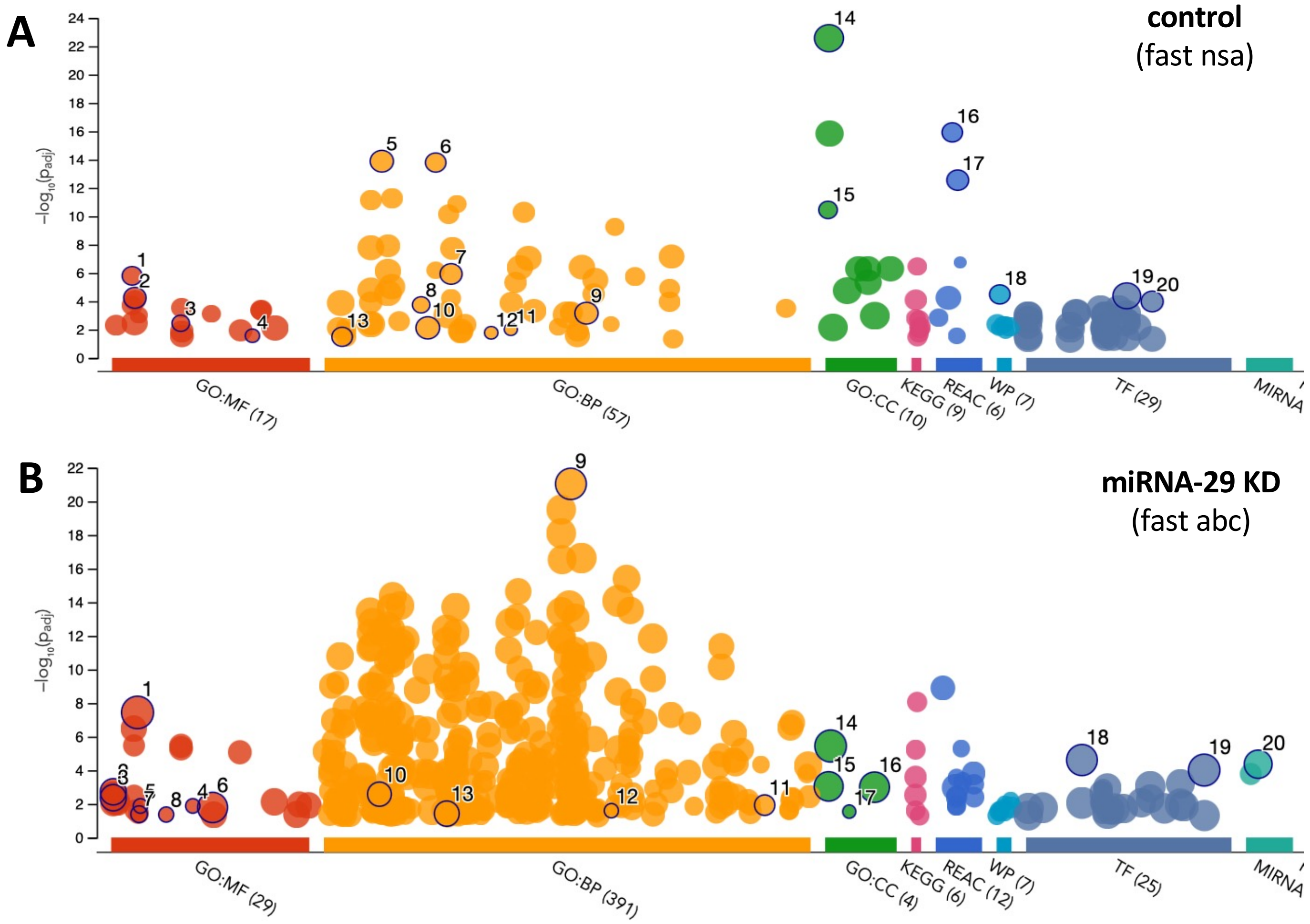

C

ID	Source	Term ID	Term Name	p_{adj} (query_1
1	GO:MF	GO:0005515	protein binding	3.629×10^{-8}
2	GO:MF	GO:0000987	cis-regulatory region sequence-specific DNA b...	1.905×10^{-3}
3	GO:MF	GO:0000981	DNA-binding transcription factor activity, RNA ...	4.306×10^{-3}
4	GO:MF	GO:0033550	MAP kinase tyrosine phosphatase activity	1.327×10^{-2}
5	GO:MF	GO:0008330	protein tyrosine/threonine phosphatase activity	1.327×10^{-2}
6	GO:MF	GO:0043167	ion binding	1.640×10^{-2}
7	GO:MF	GO:0008157	protein phosphatase 1 binding	4.124×10^{-2}
8	GO:MF	GO:0017017	MAP kinase tyrosine/serine/threonine phospha...	4.316×10^{-2}
9	GO:BP	GO:0050896	response to stimulus	9.129×10^{-22}
10	GO:BP	GO:0008015	blood circulation	2.650×10^{-3}
11	GO:BP	GO:1904659	glucose transmembrane transport	1.188×10^{-2}
12	GO:BP	GO:0061518	microglial cell proliferation	2.554×10^{-2}
13	GO:BP	GO:0030029	actin filament-based process	3.805×10^{-2}
14	GO:CC	GO:0005737	cytoplasm	3.607×10^{-6}
15	GO:CC	GO:0005576	extracellular region	9.139×10^{-4}
16	GO:CC	GO:0071944	cell periphery	1.022×10^{-3}
17	GO:CC	GO:0033093	Weibel-Palade body	2.887×10^{-2}
18	TF	TF:M07040_1	Factor: GKLF; motif: NNRRGRRNGNSNNN; mat...	2.459×10^{-5}
19	TF	TF:M12354	Factor: ZNF37A; motif: CCYYGGCTCCNTSCCMN	9.785×10^{-5}
20	MIRNA	MIRNA:hsa-mi...	hsa-miR-335-5p	4.350×10^{-5}

Thiagarajan et al. Fig. S6

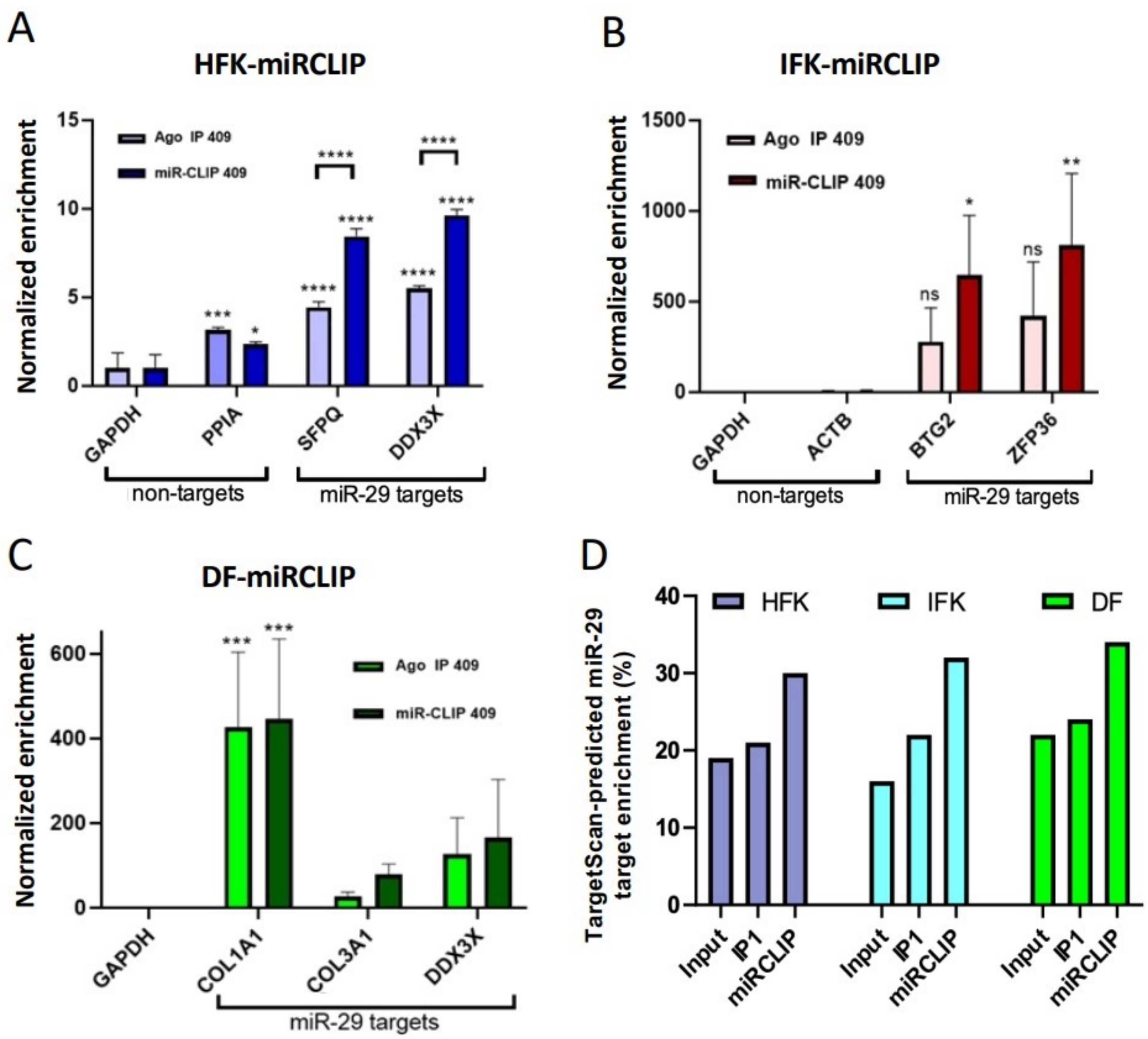

Thiagarajan et al. Fig. S7

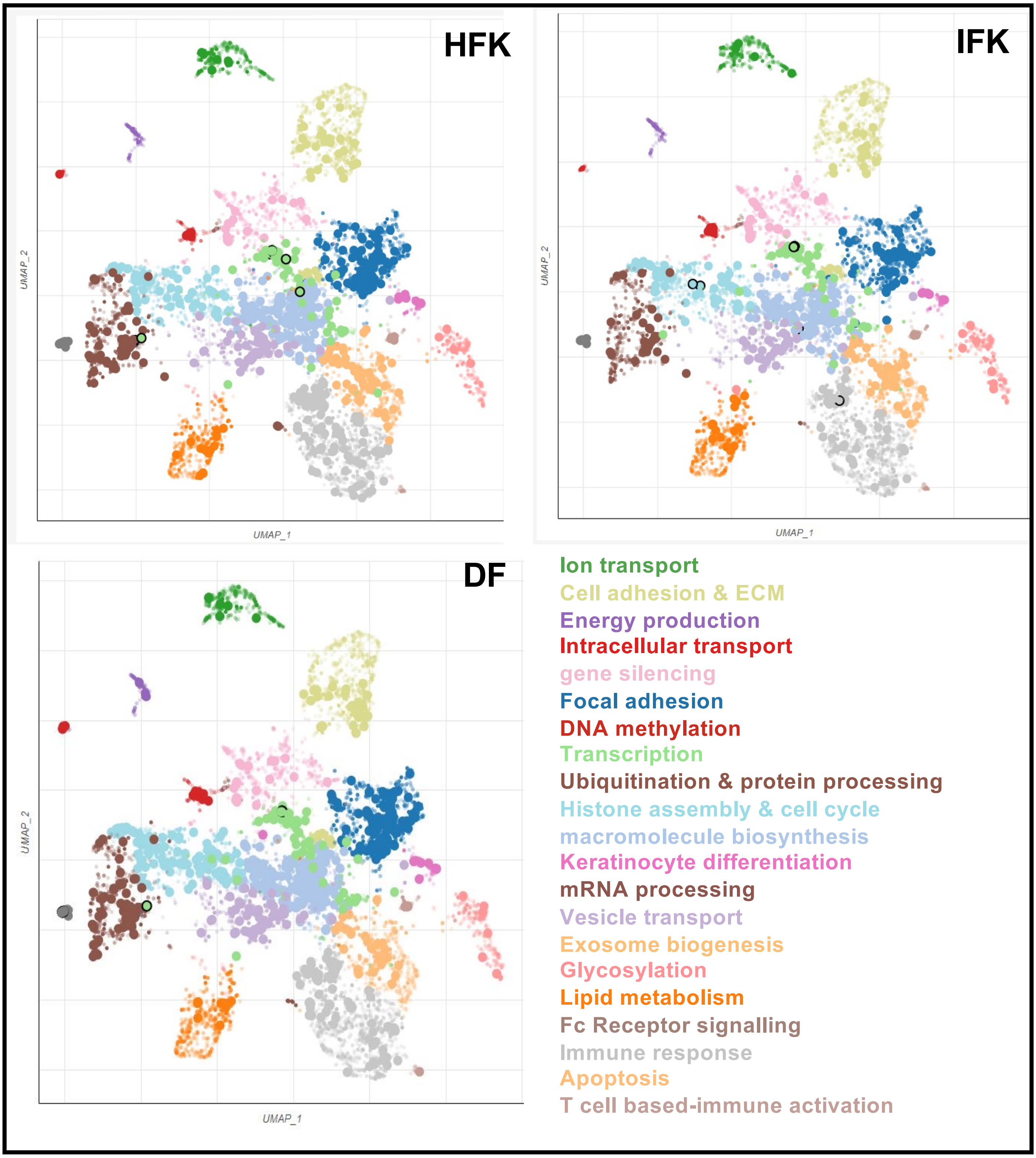

Thiagarajan et al. Fig. S8

Target names	miTG score	No. of sites
FERMT2	0.987493799	1
COL4A1	0.999996662	2
COL4A2	0.872672156	3
SPARC	0.990675772	3

FERMT2-miRNA-29 interaction

```
5'UUGC    UUACAA  U UAU         3'
      GCUG      UC G   UGGUGCUA  FERMT2 3'UTR
      .|.|      || |   ||||||||
      UGGC      AG C   ACCACGAU  miR-29a-3p
3'   U    UAA     U U           5'
```

SPARC-miRNA-29 interactions

```
5'UUGUUGUUCU  U        CAA      3'
           GCC GG   AGA   GGUGCUA  SPARC 3' UTR
           .|| |.   |||   |||||||
           UGG CU   UCU   CCACGAU  miR-29a-3p
3'        U      AAAG   A       5'

5'AGAUU   G   AUA  UUAAC        3'
       UAA UGA   CA       GGUGCUA  SPARC 3' UTR
       ||| .||   ||       |||||||
       AUU GCU   GU       CCACGAU  miR-29a-3p
3'        G   AAA  CUA          5'

5'AGUAGGAGA     CC   UU         3'
          GAUUGG  CA   UGGUGCUG  SPARC 3' UTR
          .|..|.  ||   |||||||.
          UUGGCU  GU   ACCACGAU  miR-29a-3p
3'        A     AAA  CU         5'
```

COL4A1-miRNA-29 interactions

```
5'GACUC    A  G   CAAC          3'
       AGCU AU UCA    AUGGUGCUA  COL4A1 3' UTR
       |.|. || |||    |||||||||
       UUGG UA AGU    UACCACGAU  miR-29a-3p
3'    A    C  A   C             5'

5'G   CCA      AAACCAAAG        3'
  AACU   UCAGA          GGUGCUA  COL4A1 3' UTR
  |||.   |||||          |||||||
  UUGG   AGUCU          CCACGAU  miR-29a-3p
3'A   CUAA     A                5'
```

COL4A2-miRNA-29 interactions

```
5'G  GC   AAGGGCCAUUU       U3'
  CG U CAGG          UGGUGCU   COL4A2 3' UTR
  || | |||.          |||||||
  GC A GUCU          ACCACGA   miR-29a-3p
3'G  U AA                   U5'

5'AGGUAC  CCAUAGGG          3'
      ACC        UCGGA GGUGCUG   COL4A2 3' UTR
      |||        ||.|| ||||||.
      UGG        AGUCU CCACGAU   miR-29a-3p
3'    U   CUAA        A       5'

5'CAAGGGAGU  U  CAA U       C3'
         GCC GG   CA UGGUGCU   COL4A2 3' UTR
         .|| |.   || |||||||
         UGG CU   GU ACCACGA   miR-29a-3p
3'      U     AAA CU        U5'
```

Figure S8. Analysis of the 'seed' sequence match in miRNA-29 targets 3'UTRs using DIANA tool.

Table S1

Gene_ID	CD97	EDNRB	ITGA9	NTN1	S100A8
ABL2	CD99	EFNA1	ITGAE	NUAK1	S100A9
AC002094.1	CDC42	EFNA5	ITGAL	OLFM4	S1PR1
ACTN1	CDH1	EFNB1	ITGAM	OLR1	S1PR3
ACTN2	CDH11	EFNB2	ITGAV	PARD3	SDC1
ACTN4	CDH13	EFNB3	ITGAX	PARVA	SDC3
ADAM12	CDH15	EGF	ITGB1	PARVB	SDC4
ADAM15	CDH16	EGFR	ITGB1BP1	PCDH10	SELE
ADAM17	CDH17	EMR2	ITGB2	PCDH17	SELL
ADAM9	CDH2	ENG	ITGB4	PDGFRA	SELP
AGT	CDH3	EPHA1	ITGB5	PDGFRB	SELPLG
AGTR1	CDH5	EPHA2	ITGB6	PDPK1	SEMA4D
AGTR2	CDH9	EPHA3	ITGB7	PDPN	SFRP1
AIMP1	CDK5	EPHA4	ITGB8	PIK3CA	SHC1
AKT1	CDON	EPHA8	JAM3	PIK3CB	SHH
ALCAM	CEACAM1	EPHB1	JUB	PIK3CD	SIRPA
ANGPT1	CHL1	EPHB2	JUP	PIK3CG	SLIT2
AOC3	CIB1	EPHB3	KIT	PIP5K1C	SORBS1
APC	CLCA2	EPHB4	KITLG	PKD1	SORBS3
ARF6	CLDN1	EPHB6	L1CAM	PLXNC1	SOX9
ARHGAP5	CLDN11	EZR	LAMA1	PODXL	SPN
AXL	CLDN4	F11R	LAMA3	POSTN	SPP1
BCAM	CLDN7	F2R	LAMA4	PPAP2B	SRC
BCAR1	CNTN1	F2RL1	LAMA5	PPARD	SRF
BCL2	COL11A1	FAT1	LAMB1	PPFIA1	SSTR2
BCL2L11	COL17A1	FBLIM1	LAMC1	PRKCA	STAB1
BCR	COL18A1	FBLN5	LAMC2	PRKCB	SULF1
BDKRB2	COL3A1	FER	LEF1	PRKCD	SYK
BMP7	COL4A1	FERMT1	LEP	PRKCE	TBP
BMPR1B	COL4A2	FERMT3	LGALS1	PRKCI	TCF7L2
BMX	COL4A3	FN1	LGALS3BP	PRKCQ	TEK
BRCA1	COL4A4	FPR1	LGALS9	PRKCZ	TGFB1
CADM1	COL4A6	FPR2	LIMS1	PRKD1	TGFB1I1
CADM3	CTGF	FZD7	LOXL2	PRKD2	TGFB2
CALCA	CTNNA1	GAPDH	LPAR1	PTK2	TGFB3
CASK	CTNNAL1	GAS6	LPAR2	PTK2B	TGFBI
CCKBR	CTNNB1	GATA1	LPAR3	PTPRC	THBS1
CCL11	CTNND1	GP1BA	LPP	PTPRD	THBS2
CCL2	CTNND2	GPER	LPXN	PTPRF	THRB
CCL21	CX3CL1	HAS2	MADCAM1	PTPRK	THY1
CCL4	CX3CR1	HCK	MAPK1	PTPRM	TLN1
CCL5	CXADR	HEPACAM	MAPK3	PVR	TLN2
CCR1	CXCL12	HPRT1	MCAM	PVRL1	TLR4
CCR2	CXCR1	HPSE	MERTK	PVRL2	TNC
CCR3	CXCR2	ICAM1	MFGE8	PVRL3	TNF
CCR5	CXCR3	ICAM2	MLLT4	PVRL4	TNFAIP6
CCR6	CXCR4	ICAM3	MSLN	PXN	TNFRSF12A
CCR7	CXCR5	IFNG	MSN	RAC1	TRIP6
CCR8	CXCR6	IGFBP7	MUC1	RAPGEF1	VCAM1
CCR9	CXCR7	IL1B	MUC16	RARA	VCAN
CD151	CYR61	IL2	MUC4	RASA1	VCL
CD164	DAG1	IL32	MYCN	RDX	VWF
CD2	DDR1	ILK	MYH9	RELN	WISP1
CD209	DDR2	INPPL1	NCAM1	RET	WISP2
CD22	DLC1	ITGA1	NEDD9	RHOA	WNT1
CD2AP	DPP4	ITGA11	NFKB1	RHOB	WNT2
CD34	DPT	ITGA2	NID1	RND3	WNT3A
CD36	DSC2	ITGA2B	NME1	ROBO1	WNT5A
CD4	DSC3	ITGA3	NME1-NME2	ROCK1	WNT7A
CD40LG	DSG1	ITGA4	NME2	ROCK2	ZAP70
CD44	DSG2	ITGA5	NRCAM	ROR2	ZYX
CD47	DSG3	ITGA6	NRP1	RPSA	
CD58	DST	ITGA7	NRP2	RXRA	
CD9	EDNRA	ITGA8			

Table S2

Cadherin-mediated cell adhesion	Cell-matrix glycoconjugates	ECM remodeling	Endothelial cell contacts by junctional mechanisms	Endothelial cell contacts by non-junctional mechanisms	Ephrin signaling	Gap junctions	Integrin inside-out signaling	Integrin-mediated cell adhesion and migration	Plasmin signaling	PLAU signaling	Tight Junctions
ACTA1	AMBP	AC002094.1	ACTB	AC002094.1	ADAM10	ACTA1	ACTB	ACTB	COL4A1	AC002094.1	ACTA1
ACTA2	CCL5	CD44	ACTG1	ACTB	CDC42	ACTA2	ACTG1	ACTG1	COL4A2	AKT1	ACTA2
ACTB	CCL8	COL1A1	ACTN1	ACTG1	EFNA1	ACTB	AGT	ACTN1	COL4A3	AKT2	ACTB
ACTC1	CD44	COL1A2	ACTN2	ACTN1	EFNA5	ACTC1	AGTR1	ACTN4	COL4A4	AKT3	ACTC1
ACTG1	CEACAM5	COL2A1	ACTN4	ACTN4	EFNB1	CAV1	CALM1	CFL1	F12	BCAR1	CAV1
CDC42	ECM1	COL3A1	CDH2	CDH2	EFNB2	GAPDH	CALM2	COL1A1	FGA	CSNK2A1	CDX1
CDH1	ELANE	COL4A1	CDH5	COL1A1	EFNB3	GJA1	CALM3	COL1A2	FGB	EGF	CDX2
CDH2	ELN	COL4A2	CLDN1	COL1A2	EPHA1	GJA4	CAMK2G	COL2A1	FGF2	EGFR	CLDN1
CDH3	FBLN1	COL4A3	CLDN3	COL4A1	EPHA2	GJA5	CIB1	COL4A1	FGFR1	ELK1	CLDN11
CDH5	GAPDH	EGFR	CLDN5	COL4A2	EPHA3	GJB1	COL1A1	COL4A2	FGG	GAPDH	CLDN18
CTNNA1	HPRT1	ERBB4	CTNNA1	COL4A3	EPHA4	GJB2	COL1A2	COL4A3	FN1	GRB2	CLDN2
CTNNB1	ITGB1	EZR	CTNNB1	COL4A4	EPHB1	GJC1	COL2A1	COL4A4	GAPDH	HGF	CLDN3
CTNND1	LGALS3	FN1	CTNND1	CTNNA1	EPHB2	HPRT1	CXCL12	CRK	HPRT1	HPRT1	CLDN4
CTTN	LGALS4	GAPDH	DSP	CTNNB1	EPHB3	OCLN	CXCR4	FN1	KDR	HRAS	CLDN5
FYN	LYVE1	HBEGF	F11R	FN1	EPHB4	PRKCA	F2	GAPDH	KLKB1	IL6ST	CLDN7
GAPDH	MMP1	HPRT1	GAPDH	GAPDH	FYN	PRKCB	F2R	GRB2	LAMA1	ILK	CLDN8
HPRT1	MMP3	IGF1	GJA1	HPRT1	GAPDH	PRKD2	FGA	HPRT1	LAMB1	ITGB1	CSDA
JUP	MMP9	IGF1R	GJA4	ITGA2	GNAI1	RP11-566K11.2	FGB	ITGA1	LAMC1	JAK1	F11R
MET	MUC1	IGF2	GJA5	ITGA3	GNAI2	SRC	FGG	ITGA2	MAP2K3	MAP2K1	GAPDH
MLLT4	NCAM1	IL8	HPRT1	ITGA5	GRB10	TBP	FN1	ITGA3	MAP2K6	MAP2K2	HPRT1
PTK2	PLG	ITGB1	JAM2	ITGA6	GRB2	TJP1	GAPDH	ITGA5	MAP3K7	MAPK1	ITGB1
PTPN11	SELE	KLK3	JUP	ITGAV	GRB7	TJP2	GNAI1	ITGB1	MAPK12	MAPK3	JAM2
PTPRF	SELL	LAMC2	OCLN	ITGB1	HPRT1	TUBA1B	GNAQ	LAMA1	MAPK13	MET	JAM3
PTPRJ	TBP	MMP1	TBP	JUP	HRAS	TUBB	GNB1	LAMB1	MAPK14	NCL	LEF1
PTPRM	TIMP2	MMP13	TJP1	LAMA4	KALRN	TUBB2C	GNB3	LAMC1	MMP13	PIK3CA	MLLT4
SRC	TNC	MMP14	TJP2	LAMB1	MAP3K7	TUBB3	HPRT1	MAPK1	PIK3CA	PIK3CB	MTDH
TBP	VCAN	MMP2	VIM	TBP	MAP4K4	TUBB4	ITGA1	MAPK3	PIK3CB	PIK3CD	MYL12A
		MMP3			MAPK8		ITGA2	MYH15	PIK3CD	PIK3R1	MYL12B
		MMP7			PAK1		ITGA2B	MYL9	PIK3R1	PLAT	MYL6B
		MMP9			PIK3CG		ITGA5	MYLK	PLAT	PLAU	MYL9
		MSN			PTK2		ITGAL	PAK1	PLAU	PLAUR	NKX2-1
		PLAT			PTPN13		ITGAV	PTK2	PLG	PLG	OCLN
		PLAU			PXN		ITGB1	PXN	PZP	PTK2	PVRL1
		PLAUR			RAC1		ITGB2	RAC1	SERPING1	RAF1	SNAI1
		PLG			RAF1		ITPR1	RASGRF1	TBP	SERPINA5	SP1
		SERPINE1			RAP1A		MAPK1	RHOA	TFPI2	SERPINB2	TBP
		SERPINE2			RASA1		MAPK3	ROCK1	TGFB1	SERPINE1	TCF7
		SPARC			RGS3		PIK3CG	ROCK2	TGFB2	SERPINE2	TCF7L1
		TBP			RHOA		PLCB1	SRC	TGFBR1	SHC1	TCF7L2
		TIMP1			SLA		PRKCE	TBP	TGFBR2	SOS1	TJP1
		TIMP2			SRC		PTK2	TLN1	TGFBR3	SRC	TJP2
		TIMP3			TBP		SRC	VCL	VEGFA	STAT1	TJP3
		VCAN			TIAM1		TBP	ZYX	XIAP	TBP	WNK4

Table S3

S.No	Gene description	Gene name	miRNA-29 ASO/control	
			log2FC	Fold change
1	carcinoembryonic antigen related cell adhesion molecule 19	CEACAM19	0.43662	1.35343
2	epithelial cell adhesion molecule	EPCAM	0.16463	1.12088
3	intercellular adhesion molecule 1	ICAM1	0.73879	1.66878
4	intercellular adhesion molecule 5	ICAM5	0.34145	1.26703
5	L1 cell adhesion molecule	L1CAM	0.39995	1.31946
6	nectin cell adhesion molecule 2	NECTIN2	0.28976	1.22244
7	nectin cell adhesion molecule 4	NECTIN4	0.38792	1.3085

Table S4

Catalog number: 4331182		
1	Hs00234160_m1	SPARC
2	Hs00164004_m1	COL1A1
3	Hs00235033_m1	FERMT2
4	Hs00943809_m1	COL3A1
5	Hs99999903_m1	ACTB
6	Hs00197750_m1	TXNIP
7	Hs00185658_m1	ZFP36
8	Hs00198887_m1	BTG2
9	Hs00361185_m1	KRT5
10	Hs01043717_m1	LAMC2
11	Hs00266237_m1	COL4A1
12	Hs05006309_m1	COL4A2
13	Hs00198887_m1	BTG2
14	Hs99999904_m1	PPIA
15	Hs00606179_m1	DDX3X
16	Hs00185658_m1	ZFP36
17	Hs01076943_m1	BCL2L11
18	Hs00958880_m1	PLAUR
19	Hs01034602_m1	PXDN
20	Hs01015816_m1	LYN
21	Hs00610590_m1	NEDD9
22	Hs00158757_m1	LOXL2
Catalog number: 4427975		
S.No	miR	Assay ID
1	hsa-miR-29a-3p	002112
2	hsa-miR-29b-3p	000413
3	hsa-miR-29c-3p	000587
4	RNU48	001006
5	snoRNA202	001232

Original Western blots

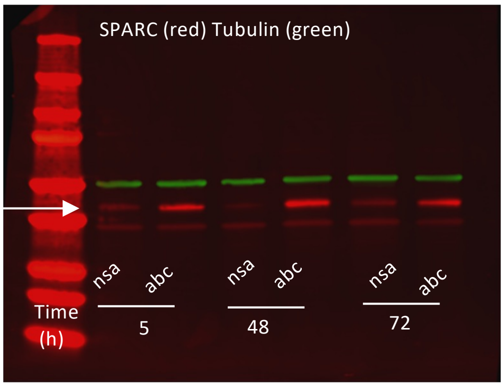

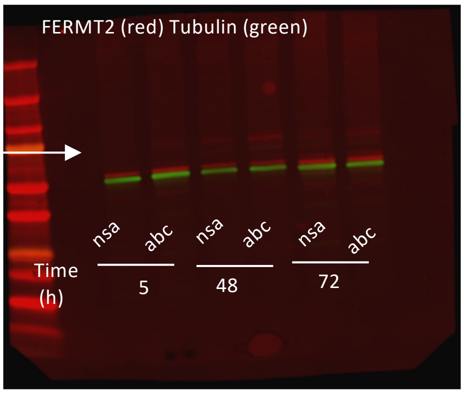

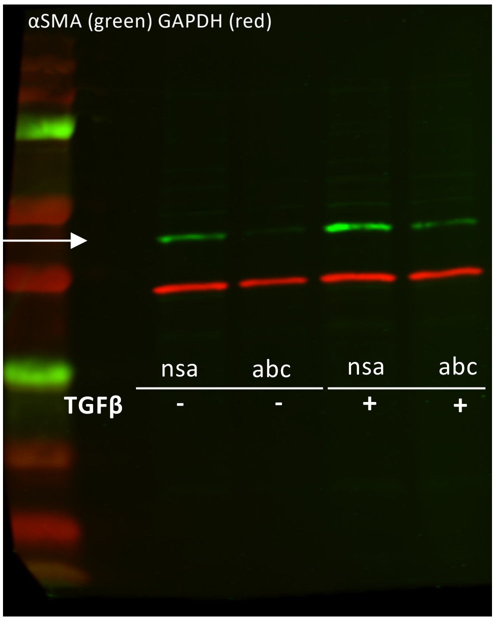

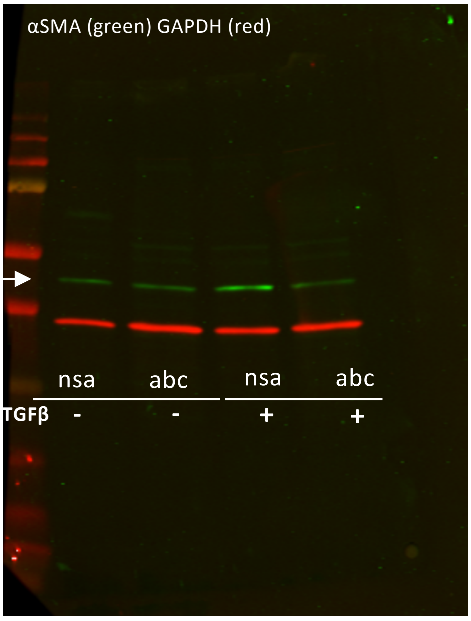

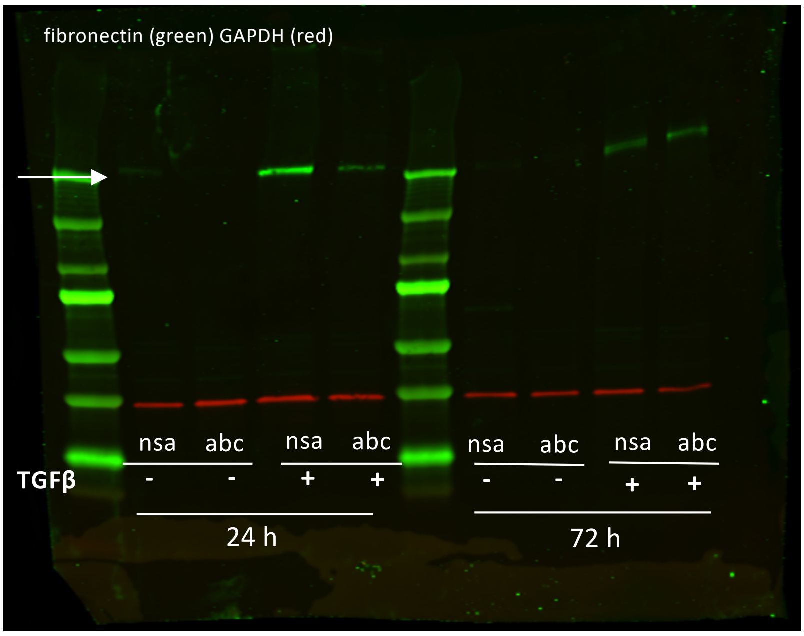

Supplement: Supplementary file 1 — Fig. S1. Gain of miRNA‐29 function results in differentiation of human keratinocytes but does not affect their proliferation. Fig. S2. miRNA‐29 regulates growth of human SE. Fig. S3. Inhibition of miRNA‐29 supports keratinocyte growth. Fig. S4. Discovering miRNA‐29‐dependent and independent mechanisms of enhanced keratinocyte adhesion. Fig. S5. miRNA‐29 regulates basal cell adhesion. Fig. S6. Validation of miRNA‐29‐CLIP in primary skin cells. Fig. S7. Functional analysis of miRNA‐29‐CLIP targetome in primary skin cells. Fig. S8. Analysis of the ‘seed’ sequence match in miRNA‐29 targets 3′UTRs using DIANA tool. Table S1. Cell adhesion pathways directly or indirectly regulated by miRNA‐29. Table S2. Cell adhesion pathways directly or indirectly regulated by miRNA‐29 further analyzed from Table S1. Table S3. Cell Adhesion Molecules. Table S4. Oligonucleotide sequences used in analyses. Data S1. Original Western blots show full size original blots scanned as described in the methods. [file FEB2-599-1795-s001.zip › !!S. figures_LT_Rev2.pdf]
