# Supplementary material for: miRNA‐29 regulates epidermal and mesenchymal functions in skin repair
Source: FEBS Lett. 2025 Apr 25;599(12):1795–817. doi: 10.1002/1873-3468.70051 (PMC12183617; doi:10.1002/1873-3468.70051)
Supplement: Supplementary file 1 — Fig. S1. Gain of miRNA‐29 function results in differentiation of human keratinocytes but does not affect their proliferation. Fig. S2. miRNA‐29 regulates growth of human SE. Fig. S3. Inhibition of miRNA‐29 supports keratinocyte growth. Fig. S4. Discovering miRNA‐29‐dependent and independent mechanisms of enhanced keratinocyte adhesion. Fig. S5. miRNA‐29 regulates basal cell adhesion. Fig. S6. Validation of miRNA‐29‐CLIP in primary skin cells. Fig. S7. Functional analysis of miRNA‐29‐CLIP targetome in primary skin cells. Fig. S8. Analysis of the ‘seed’ sequence match in miRNA‐29 targets 3′UTRs using DIANA tool. Table S1. Cell adhesion pathways directly or indirectly regulated by miRNA‐29. Table S2. Cell adhesion pathways directly or indirectly regulated by miRNA‐29 further analyzed from Table S1. Table S3. Cell Adhesion Molecules. Table S4. Oligonucleotide sequences used in analyses. Data S1. Original Western blots show full size original blots scanned as described in the methods. [file FEB2-599-1795-s001.zip › Supporting Information.docx]

**Supporting Information**

**Figure S1. Gain of miRNA-29 function results in differentiation of human keratinocytes but does not affect their proliferation.** **(A)** Human wound explants stained with miRNA-29a/b probes and scrambled control using FISH and counterstained with DAPI (nuclei) and Laminin-5 (basal membrane, where present). Wound site is labelled W, epidermis – E, dermis – D. Scale bar 20μm. **(B)** Quantification of miRNA-29 FISH signal in two human wound explants and in **(C)** unwounded skin (s). FU – fluorescence units. N=2 (wound explants or skin samples), n=5 (areas of epidermis), **P* < 0.05, ****P* < 0.001, *****P* < 0.0001, two-way ANOVA followed by Tukey multiple comparison analysis.

**(D)** Human primary keratinocytes transfected with miRNA-29a/b/c anti-sense oligonuclotides (abc) or non-specific oligos (nsa) and grown in 3D cultures at air-liquid interface for seven days. Scale bar = 500μm and 50μm. **(E)** Human skin equivalents (SE) grown in 3D cultures at air-liquid interface for six days, stained with the markers of early differentiation K10. The border between epidermis and the dermal scaffold is stained with collagen IV (COL4A1). Arrows indicate early expression of K10 in the basal layer of keratinocytes transfected with miRNA-29a/b mimic (abm), whereas in the SE transfected with a non-specific mimic (nsm) and in normal human skin K10 expression starts at the immediate suprabasal layer. The basal layer of the control (nsm) sample thus remains K10-free and only shows nuclear DAPI staining. Scale bar = 50μm. **(F)** Quantification of K10 in basal vs. suprabasal epidermis in SE grown for six days and **(G)** for 11 days and in the normal human skin. **P* < 0.05, **P<0.01, two-way ANOVA followed by Šídák’s multiple comparison analysis. **(H)** Epidermal thickness of human skin equivalents transfected with non-specific mimic (nsm), non-specific inhibitor (nsa), miRNA-29 mimic (abm), miRNA-29 inhibitor (abc) and shown in **Figure 1E** and **Figure S1D.** Staining for the same in normal human skin is shown as an additional control. ****P* < 0.001 or *****P* < 0.0001, one-way ANOVA followed by Tukey multiple comparison analysis. **(I)** Quantification of BrdU-positive keratinocytes in full-length SE epithelium expressed as % of all cells. BrdU was measured in response to transfections with non-specific mimic, non-specific inhibitor, miRNA-29 mimic, miRNA-29 inhibitor. Error bars indicate standard deviation of the mean.

**Figure S2. miRNA-29 regulates growth of human SE.** **(A-C)** Immortalised human keratinocytes were transfected miRNA-29 inhibitors (abc) or non-specific antisense (nsa) or miRNA-29 mimics (abm) or non-specific sense oligo (nsm) and grown as SE for 6 days and 11 days. Scale bar = 100μm. Epidermal thickness of quantified using Image J. *****P* < 0.0001, ns- not significant, one-way ANOVA followed by Tukey multiple comparison analysis. **(D)** Total RNA was isolated from human SE after day 6 and day 11 in culture, and miRNA-29 levels were measured by TaqMan assays. **P < 0.01, two-way ANOVA followed by Šídák’s multiple comparison. **(E)** miRNA-29 levels were measured in primary human keratinocytes after two transfections with miRNA-29 inhibitors (abc) or non-specific antisense (nsa), followed by cell adhesion assays in the absence of growth factors shown in **Figure 3D**. **P* < 0.05, ***P* < 0.01, one-way ANOVA followed by Tukey multiple comparison analysis. Error bars indicate standard deviation of the mean.

**Figure S3.** **Inhibition of miRNA-29 supports keratinocyte growth.** **(A)** Human primary keratinocytes were transfected twice with Cy3- tagged miRNA-29 anti-sense oligos (ASO) inhibiting miRNA-29a/b/c (abc) or non-specific inhibitors (nsa). Cell number was determined every day from day 1 to 8 (except day 4) using PrestoBlue, n=3; ****p<0.0001, two-way ANOVA. Day 4 was chosen to visualize cells in red channel and so, the Presto blue staining could not be performed on the day as it interferes with visualization. The growth of control (non-specific) keratinocytes slowed down when they reached confluence at day 3, pointing to unspecific cytotoxicity induced by transfection of ASO. The terminal differentiation and death at day 5 resulted in a rapid decline of cell numbers by day 6, which is sooner than the end of culture for untransfected human keratinocytes [49]. In contrast to this, oligos that bind and inhibit miRNA-29 allowed keratinocytes to stay attached and alive beyond day 5. **(B)** Representative images of the cells from each treatment after the last transfection showing signs of keratinocyte growth arrest in the control (nsa) inhibitors-treated samples. **(C)** Relative levels of miRNA-29a/b was measured after the 2^nd^ transfection and at the end of day 4 and day 8 to assess the efficiency of the inhibitors. 2-way ANOVA was performed followed by Šídák’s multiple comparisons test to ascertain the statistical significances between treatments. Error bars indicate standard deviation of the mean.

**Figure S4. Discovering miRNA-29-dependent and independent mechanisms of enhanced keratinocyte adhesion. (A)** schematic overview of experimental set-up and analysis of the mRNAs affected by inhibition of miRNA-29 in fast vs. slow adhering primary human keratinocytes. **(B)** Principal Component Analysis (PCA) using the rlog data for the top 500 most variable genes shows complete separation of RNAs that change expression in fast and slow adhering cells in response to transfection with the control (nsa) and miRNA-29 (abc) anti-sense inhibitors.

**Figure S5. miRNA-29 regulates basal cell adhesion.** EnrichR-based analysis of molecular functions (MF), biological processes (BP), cellular compartments (CC), transcription factor (TF) and miRNAs upregulated upon fast adhesion in control cells **(A)** and miRNA-29 KD cells **(B). (C)** lists the results in order of significance in MF, BP, CC, TF, and miRNA categories.

**Figure S6. Validation of miRNA-29-CLIP in primary skin cells.** Enrichment of predicted miRNA-29 targets (as predicted in TargetScan tool) through miRNA-CLIP using the 409 probe in the first step of immunoprecipitation (IP1) and in the final step of miRNA-CLIP protocol, determined in human follicular keratinocytes (HFK), interfollicular keratinocytes (IFK) and dermal fibroblasts (DF) using qPCR **(A-C)** and TargetScan **(D)** analyses. *P < 0.05 or **P < 0.01 or ****P* < 0.001 or *****P* < 0.0001, two-way ANOVA, Šídák's multiple comparisons test. Error bars indicate bidirectional standard deviation.

**Figure S7. Functional analysis of miRNA-29-CLIP targetome in primary skin cells.** Scatterplot of all terms in the GO_Biological_Processes that are represented in the miRNA-29 targetomes of human follicular keratinocytes (HFK) **(A),** interfollicular keratinocytes (IFK) **(B)** and dermal fibroblasts (DF) **(C)** using EnrichR Appyter. Each point represents a GO term from the targetome. The terms are plotted based on the first two Uniform Manifold Approximation and Projection (UMAP) dimensions. Terms are coloured by automatically identified clusters computed with the Leiden algorithm applied to the Term frequency-inverse document frequency (TF-IDF) values. Points are plotted on the first two UMAP dimensions. The darker and larger the point, the more significantly enriched the term.

**Figure S8.** **Analysis of the ‘seed’ sequence match in miRNA-29 targets 3’UTRs using DIANA tool.**

**Table S1. Cell adhesion pathways directly or indirectly regulated by miRNA-29.** Cell adhesion pathways and the gene targets were selected from BioRad assays.

**Table S2. Cell adhesion pathways directly or indirectly regulated by miRNA-29 further analysed from Table S1.** Pathways that function in basal keratinocyte adhesion have green subheadings. Genes that showed significant up-regulation in the fast population of keratinocytes following inhibition of miRNA-29 with inhibitors are highlighted in yellow.

**Table S3. Cell Adhesion Molecules.** Cell adhesion molecules that showed significant up-regulation in the fast population of miRNA-29 inhibitor-treated keratinocytes.

**Table S4. Oligonucleotide sequences used in analyses.**

**Original Western blots** show full size original blots scanned as described in the methods.
